# Weighted composition operators on the logarithmic Bloch-Orlicz space

**DOI:** 10.1371/journal.pone.0303336

**Published:** 2024-05-28

**Authors:** Hang Zhou

**Affiliations:** Department of Information Technology and Engineering, Guangzhou College of Commerce, Guangzhou, P.R. China; Karlstad University: Karlstads Universitet, SWEDEN

## Abstract

The boundedness and compactness of weighted composition operators on the logarithmic Bloch-Orlicz space $B_{\text{log}}^{\varphi }$ are investigated in this paper.

## 1 Introduction

Suppose that S(D) is the set of all analytic self-maps of the unit disk D (the analytic maps from D to itself). Let H(D) be, as usual, the collection of all analytic functions on D. The composition operator, induced by an analytic self-map of the unit disk *φ*, could be defined as

$$C_{\phi }f=f\circ \phi ,f\in H\left(D\right).$$

For ψ∈H(D), the weighted composition operator could be defined as

$$\psi C_{\phi }f=\psi \cdot f\circ \phi ,f\in H\left(D\right).$$

The study of composition operators and weighted composition operators has been established for over 6 decades. Many mathematicians aimed at the research of (weighted) composition operators on spaces of analytic functions on D or on some high-dimension domains (e.g., the unit ball of $C^{n}$, the unit polydisk of $C^{n}$). We refer to reference [1] for studying the history of composition operators acting on different spaces of analytic functions.

As one of the classical space of analytic functions on D, the Bloch space, is defined as

$$B={\left\{f\in H\left(D\right):\|f\right\|}_{B}=\underset{z\in D}{\text{sup}}{\left(1-|z\right|}^{2})|f^{\prime }\left(z\right)\left|<\infty \right\}.$$

Importantly, B is maximal among all Möbius-invariant spaces of analytic functions and B turns to a complete normed linear space endowed with the norm

$${\left\|f\right\|}_{1}=\left|f\left(0\right)\right|+{\left\|f\right\|}_{B}.$$

The logarithmic Bloch space $B_{log}$ is defined as

$$\begin{array}{c} B_{log}={\left\{f\in H\left(D\right):\|f\right\|}_{B_{log}}=\underset{z\in D}{\text{sup}}{\left(1-|z\right|}^{2})\text{log}\frac{2}{1-|z|}|f^{\prime }\left(z\right)\left|<\infty \right\}. \end{array}$$

It is a Banach space endowed with the norm ${\left\|f\right\|}_{log}=\left|f\left(0\right)\right|+{\left\|f\right\|}_{B_{log}}$.

The *μ*–Bloch space $B_{\mu }$ is defined as

$$\begin{array}{c} B_{\mu }={\left\{f\in H\left(D\right):\|f\right\|}_{B_{\mu }}=\underset{z\in D}{\text{sup}}\;\mu \left(z\right)|f^{\prime }\left(z\right)\left|<\infty \right\}, \end{array}$$

where the weight function *μ*(*z*) is a positive continuous function in D. Then $B_{\mu }$ is a Banach space endowed with the norm ${\left\|f\right\|}_{\mu }=\left|f\left(0\right)\right|+{\left\|f\right\|}_{B_{\mu }}$.

Recall that a Young’s function *φ*: [0, ∞) → [0, ∞) is a strictly increasing convex function such that *φ*(0) = 0 and $\underset{x\to \infty }{\text{lim}}\;\varphi \left(x\right)=\infty$.

In the recent years, the boundedness and compactness of composition operators among different Bloch(-type) spaces were studied (see, e.g., [2–17] and the references therein). Moreover, as the study of Hardy-Orlicz space and Bergman-Orlicz space (see, e.g., [8–14]), the Bloch-Orlicz space $B^{\varphi }$ was defined as a generalization of B.

It was firstly defined in [15],

$$\begin{array}{c} B^{\varphi }=\{f\in H\left(D\right):\underset{z\in D}{\text{sup}}{\left(1-|z\right|}^{2})\varphi (\lambda |f^{\prime }\left(z\right)|)<\infty \} \end{array}$$

for some λ > 0 depending of *f*, where *φ* is the Young’s function. Basic properties of the convex function imply

φ(st)≤sφ(t),s<1,t>0,

φ(st)≥sφ(t),s>1,t>0

and

$$\begin{array}{c} \varphi^{-1}\left(st\right)\leq s\varphi^{-1}\left(t\right),s>1,t>0. \end{array}$$ (1.1)

We can assume without loss of generality that *φ*^−1^ is differentiable (as the authors did in [15]). Suppose that

$$\begin{array}{c} S_{\varphi }\left(f\right)≔\underset{z\in D}{\text{sup}}{\left(1-|z\right|}^{2})\varphi (|f\left(z\right)|), \end{array}$$

then

$$\begin{array}{c} {\left\|f\right\|}_{\varphi }=\text{inf}\left\{k>0:S_{\varphi }\left(\frac{f^{\prime }}{k}\right)\leq 1\right\} \end{array}$$

defines a semi-norm for $B^{\varphi }$. In this way, $B^{\varphi }$ is a Banach space endowed with the norm

$${\left\|f\right\|}_{B^{\varphi }}≔\left|f\left(0\right)\right|+{\left\|f\right\|}_{\varphi }.$$

In this paper, we generalize the Bloch-Orlicz space to the logarithmic Bloch-Orlicz space. Note that it is a generalization of the logarithmic Bloch space $B_{log}$. The logarithmic Bloch-Orlicz space $B_{log}^{\varphi }$ is defined as follows,

$$\begin{array}{c} B_{\text{log}}^{\varphi }=\{f\in H\left(D\right):\underset{z\in D}{\text{sup}}{\left(1-|z\right|}^{2})\text{log}\frac{2}{1-|z|}\varphi (\lambda |f^{\prime }\left(z\right)|)<\infty \} \end{array}$$

for some λ > 0 depending of *f*, where *φ* is the Young’s function and *φ*^−1^ is differentiable. Moreover,

$$\begin{array}{c} {\left\|f\right\|}_{\varphi ,\text{log}}=\text{inf}\left\{k>0:S_{\varphi ,\text{log}}\left(\frac{f^{\prime }}{k}\right)\leq 1\right\} \end{array}$$

defines a semi-norm for $B_{\text{log}}^{\varphi }$, where

$$\begin{array}{c} S_{\varphi ,\text{log}}\left(f\right)≔\underset{z\in D}{\text{sup}}{\left(1-|z\right|}^{2})\text{log}\frac{2}{1-|z|}\varphi \left(|f\left(z\right)|\right). \end{array}$$

Thus, $B_{\text{log}}^{\varphi }$ becomes a Banach space endowed with the norm

$${\left\|f\right\|}_{B_{\text{log}}^{\varphi }}≔\left|f\left(0\right)\right|+{\left\|f\right\|}_{\varphi ,\text{log}}.$$

This paper is organized as follows: in Section 2 we recall some basic facts on the logarithmic Bloch-Orlicz space. In Section 3 we investigate the boundedness of weighted composition operators on the logarithmic Bloch-Orlicz space. Moreover, in Section 4 we investigate the compactness of weighted composition operators on the logarithmic Bloch-Orlicz space.

We say that φ∈U if there exists a constant *M*_*φ*_ > 1 such that

$$\varphi \left(st\right)\leq M_{\varphi }s\varphi \left(t\right),s>1,t>0,$$

which also implies that

$$s\varphi^{-1}\left(t\right)\leq \varphi^{-1}\left(st\right)\leq M_{\varphi }s\varphi^{-1}\left(t\right),s<1,t>0$$

and

$$s\varphi^{-1}\left(t\right)\geq \varphi^{-1}\left(st\right)\geq \frac{1}{M_{\varphi }}s\varphi^{-1}\left(t\right),s>1,t>0.$$

## 2 Preliminaries

In this section, we present several basic conclusions for the study of $B_{\text{log}}^{\varphi }$.

**Proposition 2.1** [16]

$$S_{\varphi ,\alpha }\left(\frac{f^{\prime }}{{\left\|f\right\|}_{B_{\text{log}}^{\varphi }}}\right)\leq S_{\varphi ,\alpha }\left(\frac{f^{\prime }}{{\left\|f\right\|}_{\varphi ,\text{log}}}\right)\leq 1$$

*holds for each* $f\in B_{\text{log}}^{\varphi }$.

*Proof*. The proof is similar with Lemma 2 in [15].

For each $f\in B_{\text{log}}^{\varphi }\backslash \left\{0\right\}$, a decreasing sequence {λ_*n*,log_}_*n*_ of positive numbers can be chosen, satisfying $\underset{n\to \infty }{\text{lim}}\lambda_{n,\text{log}}={\left\|f\right\|}_{\varphi ,\text{log}}$ and $S_{\varphi ,\text{log}}\left(\frac{f^{\prime }}{\lambda_{n,\text{log}}}\right)\leq 1.$ For any positive integer n∈N, let $S_{n}≔S_{\varphi ,\text{log}}\left(\frac{f^{\prime }}{\lambda_{n,\text{log}}}\right)$. Observe that {*S*_*n*_} is increasing and bounded and hence there exists a real number $S^{\prime }\in R$ satisfying $\underset{n\to \infty }{\text{lim}}S_{n}=S^{\prime }.$ It follows that $S^{\prime }=\underset{n\in N}{\text{sup}}\left\{S_{n}\right\}\leq 1.$ Observe that

$$S_{n}=S_{\varphi ,\text{log}}\left(\frac{f^{\prime }}{\lambda_{n,\text{log}}}\right)\leq S_{\varphi ,\text{log}}\left(\frac{f^{\prime }}{{\left\|f\right\|}_{\varphi ,\text{log}}}\right)≔S.$$

Thus we have *S*′ ≤ *S*. Moreover,

$${\left(1-|z\right|}^{2})\text{log}\frac{2}{1-|z|}\varphi \left(\frac{|f^{\prime }\left(z\right)|}{\lambda_{n,\text{log}}}\right)\leq S_{\varphi ,\text{log}}\left(\frac{f^{\prime }}{\lambda_{n,\text{log}}}\right)\leq S^{\prime }\leq 1$$

holds for each z∈D and n∈N. Taking limit as *n* → ∞, the above inequality becomes

$${\left(1-|z\right|}^{2})\text{log}\frac{2}{1-|z|}\varphi \left(\frac{|f^{\prime }\left(z\right)|}{{\left\|f\right\|}_{\varphi ,\text{log}}}\right)\leq S_{\varphi ,\text{log}}\left(\frac{f^{\prime }}{\lambda_{n,\text{log}}}\right)\leq S^{\prime }\leq 1$$

for each z∈D, which is equivalent to say that

$$S=\underset{z\in D}{\text{sup}}{\left(1-|z\right|}^{2})\text{log}\frac{2}{1-|z|}\varphi \left(\frac{|f^{\prime }\left(z\right)|}{{\left\|f\right\|}_{\varphi ,\text{log}}}\right)\leq 1.$$

This completes the proof.

**Remark 2.2** *The inequality*

$$\begin{array}{c} \left|f\left(z\right)\right|\leq M_{\varphi }\varphi^{-1}\left(1\right)\left(2+\text{log}\;\text{log}\frac{2}{1-|z|}\right){\left\|f\right\|}_{B_{\text{log}}^{\varphi }} \end{array}$$ (2.1)

*holds for all* $f\in B_{\text{log}}^{\varphi }$ *and* z∈D *by Lemma 2.1, where M*_*φ*_ > 1 *is a constant only dependent of φ. In fact, a simple estimation shows that*

$$\begin{array}{cc} \left|f(z)-f(tz)\right| & =|z\int_{t}^{1}f^{\prime }{\left(zt\right)dt|\leq \|f\|}_{\varphi ,\text{log}}\int_{t}^{1}\varphi^{-1}\left(\frac{1}{{\left(1-|zt\right|}^{2})\text{log}\frac{2}{1-|zt|}}\right)dt\\ & \leq M_{\varphi }\varphi^{-1}\left(1\right){\left\|f\right\|}_{\varphi ,\text{log}}\int_{t}^{1}\frac{1}{1-{\left|zt\right|}^{2}}\text{log}\frac{2}{1-|zt|}dt\\ & \leq M_{\varphi }\varphi^{-1}\left(1\right)\text{log}\frac{\text{log}\frac{2}{1-|z|}}{\text{log}\frac{2}{1-|tz|}}{\left\|f\right\|}_{\varphi ,\text{log}}, \end{array}$$

*where more details can be found in* [17]. *The inequality above also implies that the evaluation functional is continuous on* $B_{\text{log}}^{\varphi }$, *where* z∈D *is fixed*.

The proposition below shows that the logarithmic Bloch-Orlicz space is isometrically equal to a *μ*–Bloch space.

**Proposition 2.3** *The logarithmic Bloch-Orlicz space is isometrically equal to a μ*_log_–*Bloch space, where*

$$\begin{array}{c} \mu_{\text{log}}\left(z\right)=\frac{1}{\varphi^{-1}\left(\frac{1}{{\left(1-|z\right|}^{2})\text{log}\frac{2}{1-|z|}}\right)}. \end{array}$$

*In other words*,

$${\left\|f\right\|}_{B_{\text{log}}^{\varphi }}=\left|f\left(0\right)\right|+\underset{z\in D}{\text{sup}}\;\mu_{\text{log}}\left(z\right)\left|f^{\prime }\left(z\right)\right|$$

*holds for each* $f\in B_{\text{log}}^{\varphi }$.

*Proof*. Deducted by Proposition 2.1, for each $f\in B_{\text{log}}^{\varphi }$ and z∈D,

$${\left(1-|z\right|}^{2})\text{log}\frac{2}{1-|z|}\varphi \left(\frac{|f^{\prime }\left(z\right)|}{{\left\|f\right\|}_{\varphi ,\text{log}}}\right)\leq 1,$$

which implies that *μ*_log_(*z*)|*f*>′(*z*)| ≤ ‖*f*‖_*φ*,log_ holds for all z∈D. Therefore, $B_{\text{log}}^{\varphi }\subset B_{\mu_{\text{log}}}$ with ${\left\|f\right\|}_{\mu_{\text{log}}}\leq {\left\|f\right\|}_{\varphi ,\text{log}}$. Conversely, for each $f\in B_{\mu_{\text{log}}}$, $\mu_{\text{log}}\left(z\right)|f^{\prime }{\left(z\right)|\leq \|f\|}_{\mu_{\text{log}}}$ holds for all z∈D, which is equivalent with

$$\frac{1}{\varphi^{-1}\left(\frac{1}{{\left(1-|z\right|}^{2})\text{log}\frac{2}{1-|z|}}\right)}|f^{\prime }{\left(z\right)|\leq \|f\|}_{\mu_{\text{log}}}.$$

It follow that $S_{\varphi ,\text{log}}\left(\frac{f^{\prime }}{{\left\|f\right\|}_{\mu_{\text{log}}}}\right)\leq 1.$ Therefore, we obtain that $B_{\mu_{\text{log}}}\subset B_{\text{log}}^{\varphi }$ with ${\left\|f\right\|}_{\varphi ,\text{log}}\leq {\left\|f\right\|}_{\mu_{\text{log}}}$. Combining what we have observed above, we complete the proof.

**Corollary 2.4** *The equivalent condition*

$$\begin{array}{c} S_{\varphi ,\text{log}}\left(f^{\prime }\right)\leq 1\Leftrightarrow {\left\|f\right\|}_{\varphi ,\text{log}}\leq 1 \end{array}$$ (2.4)

*holds for each* $f\in B_{\text{log}}^{\varphi }$ .

*Proof*. The sufficiency part is obvious. The necessity is deducted by Proposition 2.1 and the estimation $S_{\varphi ,\text{log}}\left(f^{\prime }\right)\leq S_{\varphi ,\text{log}}\left(\frac{f^{\prime }}{{\left\|f\right\|}_{\varphi ,\text{log}}}\right)\leq 1$.

For two real numbers *A* and *B*, we say *A* ≲ *B* if there exists a constant *C* ≠ 0 such that *A* ≤ *CB*, by which the complexity of all constants appearing is simplified.

## 3 Boundedness of weighted composition operator on $B_{\text{log}}^{\varphi }$

In this section we investigate the boundedness of weighted composition operators on the logarithmic Bloch-Orlicz space under the condition φ∈U(this is an unexpected hypothesis). However, for those Young’s funtion φ∉U, the boundedness of weighted composition operators on the logarithmic Bloch-Orlicz space remains to be an open question.

The first lemma contains some trivial but complicated calculations, which will be used in the proof in this section.

**Lemma 3.1** *Let*

$$g_{t}\left(z\right)=\frac{\left(1-|z|\right)\text{log}\frac{2}{1-|z|}}{\left(1-|tz|\right)\text{log}\frac{2}{1-|tz|}},$$

*where t* ∈ [0, 1] *and* z∈D, *then* |*g*_*t*_(*z*)| < 2.

Basic properties of the auxiliary functions entailed to the proof of the boundedness of weighted composition operator *ψC*_*ϕ*_ are described in the next lemma.

**Lemma 3.2** *For* a∈D , $n\in N^{+}$ *and* φ∈U, *suppose that*

$$p_{a}\left(z\right)=2\;\text{log}\;\text{log}\frac{4}{1-\bar{\phi (a)}z}-\frac{1}{\text{log}\;\text{log}\frac{4}{1-{\left|\phi \left(a\right)\right|}^{2}}}{\left(\text{log}\;\text{log}\frac{4}{1-\bar{\phi (a)}z}\right)}^{2}$$

*and*

$$q_{a}\left(z\right)=\varphi^{-1}\left(\frac{1}{{\left(1-|\phi \left(a\right)\right|}^{2})\text{log}\frac{2}{1-|\phi (a)|}}\right)\frac{-(1-|\phi \left(a\right){|}^{2})}{\bar{\phi (a)}}\text{log}\left(1-\bar{\phi (a)}z\right),$$

*where* z∈D. *Then the auxiliary functions p_a_ and q_a_ have the properties as follows*:

*(i) the auxiliary function p_a_ belongs to the logarithmic Bloch-Orlicz space* $B_{\text{log}}^{\varphi }$ *with* $\underset{a\in D}{\text{sup}}{\left\|p_{a}\right\|}_{B_{\text{log}}^{\varphi }}≲1$ .

*(ii) the auxiliary function q_a_ belongs to the logarithmic Bloch-Orlicz space* $B_{\text{log}}^{\varphi }$ *with* $\underset{a\in D}{\text{sup}}{\left\|q_{a}\right\|}_{B_{\text{log}}^{\varphi }}≲1$ .

*Proof*. As easy calculation shows,

$$p_{a}^{\prime }\left(z\right)=\frac{2\bar{\varphi (a)}}{\left(1-\bar{\varphi (a)}z\right)\text{log}\frac{4}{1-\bar{\varphi (a)z}}}-2\frac{\text{log}\;\text{log}\frac{4}{1-\bar{\varphi (a)}z}}{\text{log}\;\text{log}\frac{4}{1-{\left|\varphi \left(a\right)\right|}^{2}}}\frac{\bar{\varphi (a)}}{\left(1-\bar{\varphi (a)}z\right)\frac{4}{1-\bar{\varphi (a)}z}}$$

and

$$q_{a}^{\prime }\left(z\right)=\varphi^{-1}\left(\frac{1}{{\left(1-|\phi \left(a\right)\right|}^{2})\text{log}\frac{2}{1-|\phi (a)|}}\right)\frac{1-{\left|\phi \left(a\right)\right|}^{2}}{|1-\bar{\phi (a)}z|}$$

Observe that

$$\begin{array}{cc} & \|q_{a}{\|}_{\varphi ,\text{log}}\\ & =\underset{z\in D}{\text{sup}}\frac{1}{\varphi^{-1}\left(\frac{1}{{\left(1-|z\right|}^{2})\text{log}\frac{2}{1-|z|}}\right)}\varphi^{-1}\left(\frac{1}{{\left(1-|\phi \left(a\right)\right|}^{2})\text{log}\frac{2}{1-|\phi (a)|}}\right)\frac{1-{\left|\phi \left(a\right)\right|}^{2}}{|1-\bar{\phi (a)}z|}\\ & \leq M_{\varphi }\underset{z\in D}{\text{sup}}\frac{{\left(1-|z\right|}^{2})\text{log}\frac{2}{1-|z|}}{|1-\bar{\phi (a)}z|\text{log}\frac{2}{|1-\bar{\phi (a)}z|}}\frac{\text{log}\frac{2}{|1-\bar{\phi (a)}z|}}{\text{log}\frac{2}{1-|\phi (a)|}}\frac{1-{\left|\phi \left(a\right)\right|}^{2}}{1-{\left|\phi \left(a\right)\right|}^{2}}\leq 4M_{\varphi }. \end{array}$$

Then we conclude that $\underset{a\in D}{\text{sup}}\;S_{\varphi ,\text{log}}\left(q_{a}^{\prime }\right)≲1.$ Further observe that, by Lemma 3.1

$$\begin{array}{cc} & \underset{z\in D}{\text{sup}}\frac{1}{\varphi^{-1}\left(\frac{1}{{\left(1-|z\right|}^{2})\text{log}\frac{2}{1-|z|}}\right)}\frac{2|\phi (a)|}{|1-\bar{\phi (a)}z|\text{log}\frac{4}{|1-\bar{\phi (a)}z|}}\\ & \leq 2\;\underset{z\in D}{\text{sup}}\frac{4C_{\phi }\left(1-|z|\right)\text{log}\frac{2}{1-|z|}}{|1-\bar{\phi (a)}z|\text{log}\frac{2}{|1-\bar{\phi (a)}z|}}≲2 \end{array}$$

and

$$\begin{array}{cc} & \underset{z\in D}{\text{sup}}\frac{1}{\varphi^{-1}\left(\frac{1}{{\left(1-|z\right|}^{2})\text{log}\frac{2}{1-|z|}}\right)}\frac{2|\phi (a)|}{|1-\bar{\phi (a)}z|\text{log}\frac{4}{|1-\bar{\phi (a)}z|}}\frac{\text{log}\;\text{log}\frac{4}{|1-\bar{\varphi (a)}z|}}{\text{log}\;\text{log}\frac{4}{1-{\left|\varphi \left(a\right)\right|}^{2}}}\\ & ≲\;\underset{z\in D}{\text{sup}}\frac{\left(1-|z|\right)\text{log}\frac{2}{1-|z|}}{|1-\bar{\phi (a)}z|\text{log}\frac{2}{|1-\bar{\phi (a)}z|}}\;≲\;2. \end{array}$$

Then we conclude that $\underset{a\in D}{\text{sup}}\;S_{\varphi ,\text{log}}\left(p_{a}^{\prime }\right)\;≲\;2$.

Though the approach we use in the proof of the boundedness is standard, it is not trivial since the collection U contains not only (almost) linear functions, it also contains the convex functions which line between the the line whose tangent is 1 and a line whose tangent is *M*_*φ*_, a positive number depending on *φ*.

**Theorem 3.3** *For* φ∈U , *the weighted composition operator ψC_ϕ_ is bounded on* $B_{\text{log}}^{\varphi }$ *if and only if*

$$\begin{array}{c} M_{1}≔\underset{z\in D}{\text{sup}}\frac{|\psi^{\prime }\left(z\right)|\left(2+\text{log}\;\text{log}\frac{2}{1-|z|}\right)}{\varphi^{-1}\left(\frac{1}{{\left(1-|z\right|}^{2})\text{log}\frac{2}{1-|z|}}\right)}<\infty \end{array}$$

*and*

$$\begin{array}{c} M_{2}≔\underset{z\in D}{\text{sup}}\frac{|\psi \left(z\right)\phi^{\prime }\left(z\right)|\varphi^{-1}\left(\frac{1}{(1-|\phi \left(z\right){|}^{2}\text{log}\frac{2}{1-|\phi (z)|}}\right)}{\varphi^{-1}\left(\frac{1}{{\left(1-|z\right|}^{2})\text{log}\frac{2}{1-|z|}}\right)}<\infty \end{array}$$

*hold*.

*Proof*. Suppose that *M*_1_ < ∞, *M*_2_ < ∞. For each $f\in B_{\text{log}}^{\varphi }\backslash \left\{0\right\}$, observe that

$$\begin{array}{cc} & S_{\varphi ,\text{log}}\left(\frac{{\left(\psi C_{\phi }f\right)}^{\prime }}{{C\|f\|}_{B_{\text{log}}^{\varphi }}}\right)\\ & \leq \underset{z\in D}{\text{sup}}{\left(1-|z\right|}^{2})\text{log}\frac{2}{1-|z|}\varphi \left(\frac{|\psi^{\prime }\left(z\right)f\left(\phi \left(z\right)\right)|}{{C\|f\|}_{B_{\text{log}}^{\varphi }}}+\frac{|\psi \left(z\right)f^{\prime }\left(\phi \left(z\right)\right)\phi^{\prime }\left(z\right)|}{{C\|f\|}_{B_{\text{log}}^{\varphi }}}\right)\\ & \leq \underset{z\in D}{\text{sup}}{\left(1-|z\right|}^{2})\text{log}\frac{2}{1-|z|}\varphi (\frac{|\psi^{\prime }\left(z\right)|M_{\varphi }\varphi^{-1}\left(1\right)\left(2+\text{log}\;\text{log}\frac{2}{1-|z|}\right){\left\|f\right\|}_{B_{\text{log}}^{\varphi }}}{{C\|f\|}_{B_{\text{log}}^{\varphi }}}\\ & \;+\frac{|\psi \left(z\right)\phi^{\prime }\left(z\right)|\phi^{-1}\left(\frac{1}{{\left(1-|\phi \left(z\right)\right|}^{2})\text{log}\frac{2}{1-|\phi (z)|}}\right){\left\|f\right\|}_{B_{\text{log}}^{\varphi }}}{{C\|f\|}_{B_{\text{log}}^{\varphi }}})\\ & \leq \underset{z\in D}{\text{sup}}{\left(1-|z\right|}^{2})\text{log}\frac{2}{1-|z|}\varphi \left(\varphi^{-1}\left(\frac{1}{{\left(1-|z\right|}^{2})\text{log}\frac{2}{1-|z|}}\right)\left(\frac{M_{\varphi }\varphi^{-1}\left(1\right)M_{1}}{C}+\frac{M_{2}}{C}\right)\right)\\ & \leq \underset{z\in D}{\text{sup}}{\left(1-|z\right|}^{2})\text{log}\frac{2}{1-|z|}\varphi \left(\varphi^{-1}\left(\frac{1}{{\left(1-|z\right|}^{2})\text{log}\frac{2}{1-|z|}}\right)\right)=1, \end{array}$$

where *C* is chosen such that *M*_*φ*_*φ*^−1^(1)*M*_1_ + *M*_2_ ≤ *C* and the second inequality is deducted by (2.1). Then we conclude that *ψC*_*ϕ*_ is bounded on $B_{\text{log}}^{\varphi }$ by

$$\|\psi C_{\varphi }{f\|}_{\varphi ,\text{log}}\leq C{\left\|f\right\|}_{B_{\text{log}}^{\varphi }}$$

and the estimation (2.1) by taking *z* = *ϕ*(0).

Conversely, if *ψC*_*ϕ*_ is bounded on $B_{\text{log}}^{\varphi }$, then there exists a constant *C* ≥ 0 such that $\|\psi C_{\phi }{f\|}_{B_{\text{log}}^{\varphi }}\leq C{\left\|f\right\|}_{B_{\text{log}}^{\varphi }}$ for each $0\neq f\in B_{\text{log}}^{\varphi }$. By Proposition 2.1 and the condition above,

$$S_{\varphi ,\text{log}}\left(\frac{{\left(\psi C_{\phi }f\right)}^{\prime }}{{C\|f\|}_{B_{\text{log}}^{\varphi }}}\right)\leq S_{\varphi ,\text{log}}\left(\frac{{\left(\psi C_{\phi }f\right)}^{\prime }}{\|\psi C_{\phi }{f\|}_{B_{\text{log}}^{\varphi }}}\right)\leq 1$$

holds for each $f\in B_{\text{log}}^{\varphi }$, which is equivalent with

$$\begin{array}{c} \underset{z\in D}{\text{sup}}{\left(1-|z\right|}^{2})\text{log}\frac{2}{1-|z|}\varphi \left(\frac{|f^{\prime }\left(\phi \left(z\right)\right)\phi^{\prime }\left(z\right)\psi \left(z\right)+f\left(\phi \left(z\right)\right)\psi^{\prime }\left(z\right)|}{{C\|f\|}_{B_{\text{log}}^{\varphi }}}\right)\leq 1. \end{array}$$ (3.1)

Taking $f_{0}\left(z\right)=1\in B_{\text{log}}^{\varphi }$, employing (3.1) we obtain that

$$\begin{array}{c} L_{1}≔\underset{z\in D}{\text{sup}}\frac{|\psi^{\prime }\left(z\right)|}{\varphi^{-1}\left(\frac{1}{{\left(1-|z\right|}^{2})\text{log}\frac{2}{1-|z|}}\right)}<\infty . \end{array}$$ (3.2)

Further taking $\hat{f_{0}}=z\in B_{\text{log}}^{\varphi }$, employing (3.1) again we obtain that

$$\frac{|\phi^{\prime }\left(z\right)\psi \left(z\right)+\phi \left(z\right)\psi^{\prime }\left(z\right)|}{\varphi^{-1}\left(\frac{1}{{\left(1-|z\right|}^{2})\text{log}\frac{2}{1-|z|}}\right)}\leq C{\left\|f\right\|}_{B_{\text{log}}^{\varphi }}$$

holds for each z∈D. It follows that

$$\begin{array}{ccc} L_{2} & ≔\underset{z\in D}{\text{sup}}\frac{|\phi^{\prime }\left(z\right)\psi \left(z\right)|}{\varphi^{-1}\left(\frac{1}{{\left(1-|z\right|}^{2})\text{log}\frac{2}{1-|z|}}\right)}\\ & \leq \underset{z\in D}{\text{sup}}\frac{|\phi \left(z\right)\psi^{\prime }\left(z\right)|}{\varphi^{-1}\left(\frac{1}{{\left(1-|z\right|}^{2})\text{log}\frac{2}{1-|z|}}\right)} & +\underset{z\in D}{\text{sup}}\frac{|\phi^{\prime }\left(z\right)\psi \left(z\right)+\phi \left(z\right)\psi^{\prime }\left(z\right)|}{\varphi^{-1}\left(\frac{1}{{\left(1-|z\right|}^{2})\text{log}\frac{2}{1-|z|}}\right)}<\infty . \end{array}$$ (3.3)

On the one hand, for each a,z∈D, suppose that

$$p_{a}\left(z\right)=2\;\text{log}\;\text{log}\frac{4}{1-\bar{\phi (a)}z}-\frac{1}{\text{log}\;\text{log}\frac{4}{1-{\left|\phi \left(a\right)\right|}^{2}}}{\left(\text{log}\;\text{log}\frac{4}{1-\bar{\phi (a)}z}\right)}^{2}.$$

Obviously, $p_{a}^{\prime }\left(\phi \left(a\right)\right)=0$ and

$$p_{a}\left(\phi \left(a\right)\right)=\text{log}\;\text{log}\frac{4}{1-{\left|\phi \left(a\right)\right|}^{2}}$$

By Lemma 3.2, we have that $p_{a}\in B_{\text{log}}^{\varphi }.$ It follows by the boundedness of *ψC*_*ϕ*_ that

$$\frac{|\psi^{\prime }\left(a\right)|\left(\text{log}\;\text{log}\frac{4}{1-{\left|\phi \left(a\right)\right|}^{2}}\right)}{\varphi^{-1}\left(\frac{1}{{\left(1-|a\right|}^{2})\text{log}\frac{2}{1-|a|}}\right)}=\frac{|{\left(\psi C_{\phi }p_{a}\right)}^{\prime }\left(a\right)|}{\varphi^{-1}\left(\frac{1}{{\left(1-|a\right|}^{2})\text{log}\frac{2}{1-|a|}}\right)}<\infty$$

and hence by *L*_1_ < ∞ we conclude that

$$M_{1}=\underset{z\in D}{\text{sup}}\frac{|\psi^{\prime }\left(z\right)|\left(2+\text{log}\;\text{log}\frac{2}{1-|\phi (z)|}\right)}{\varphi^{-1}\left(\frac{1}{{\left(1-|z\right|}^{2})\text{log}\frac{2}{1-|z|}}\right)}<\infty .$$

On the other hand, for each a,z∈D, we define

$$q_{a}\left(z\right)=\varphi^{-1}\left(\frac{1}{{\left(1-|a\right|}^{2})\text{log}\frac{2}{1-|a|}}\right)\frac{-(1-|a{|}^{2})}{\bar{a}}\text{log}\left(1-\bar{a}z\right),$$

By Lemma 3.2, $q_{a}\in B_{\text{log}}^{\varphi }.$ Obviously,

$$q_{a}^{\prime }\left(\phi \left(a\right)\right)=\phi^{-1}\left(\frac{1}{{\left(1-|\phi \left(a\right)\right|}^{2})\text{log}\frac{2}{1-|\phi (a)|}}\right).$$

For each a∈D, we have

$$\begin{array}{cc} & \mu_{\text{log}}\left(a\right)\varphi^{-1}\left(\frac{1}{{\left(1-|\phi \left(a\right)\right|}^{2})\text{log}\frac{2}{1-{\left|\phi \left(a\right)\right|}^{2}}}\right)\left|\psi \left(a\right)\phi^{\prime }\left(a\right)\right|\\ & \leq \|\psi C_{\phi }q_{a}{\|}_{B_{\text{log}}^{\varphi }}+\mu_{\text{log}}\left(a\right)\left|\psi^{\prime }\left(a\right)q_{a}\left(\phi \left(a\right)\right)\right|\\ & \leq \|\psi C_{\phi }q_{a}{\|}_{B_{\text{log}}^{\varphi }}+\mu_{\text{log}}\left(a\right)|\psi^{\prime }\left(a\right)|\left(2+\text{log}\;\text{log}\frac{2}{1-|\phi (a)|}\right){\left\|q_{a}\right\|}_{B_{\text{log}}^{\varphi }}\\ & \leq \|\psi C_{\phi }q_{a}{\|}_{B_{\text{log}}^{\varphi }}+M_{1}{\left\|q_{a}\right\|}_{B_{\text{log}}^{\varphi }}<\infty . \end{array}$$

Then we conclude that

$$M_{2}≔\underset{z\in D}{\text{sup}}\frac{|\psi \left(z\right)\phi^{\prime }\left(z\right)|\varphi^{-1}\left(\frac{1}{(1-|\phi \left(z\right){|}^{2}\text{log}\frac{2}{1-|\phi (z)|}}\right)}{\varphi^{-1}\left(\frac{1}{{\left(1-|z\right|}^{2})\text{log}\frac{2}{1-|z|}}\right)}<\infty .$$

Combining what we have observed above, we complete the proof.

## 4 Compactness of weighted composition operator on $B_{\text{log}}^{\varphi }$

In this section we investigate the compactness of weighted composition operators on the logarithmic Bloch-Orlicz space, where the approach we use in the proof within is standard (see, e.g., [16]).

The first lemma contains some trivial but complicated calculations, which will be used in the proof of the compactness of weighted composition operator on $B_{\text{log}}^{\varphi }$. Moreover, it can be proved in a similar way with Lemma 3.2.

**Lemma 4.1** *For* a∈D , $n\in N^{+}$ *and* φ∈U, *suppose that*

$${\tilde{p}}_{a}\left(z\right)=\frac{3{\left(\text{log}\;\text{log}\frac{4}{1-\bar{\varphi (a)}z}\right)}^{2}}{\text{log}\;\text{log}\frac{4}{1-{\left|\phi \left(a\right)\right|}^{2}}}-\frac{2{\left(\text{log}\;\text{log}\frac{4}{1-\bar{\varphi (a)}z}\right)}^{3}}{{\left(\text{log}\;\text{log}\frac{4}{1-{\left|\varphi \left(a\right)\right|}^{2}}\right)}^{2}}$$

*where* z∈D. *Then the auxiliary function* ${\tilde{p}}_{a}$ *belongs to the logarithmic Bloch-Orlicz space* $B_{\text{log}}^{\varphi }$ *with* $\underset{a\in D}{\text{sup}}{\left\|{\tilde{p}}_{a}\right\|}_{B_{\text{log}}}≲1$.

**Theorem 4.2** *For* φ∈U , *the weighted composition operator ψC*_*ϕ*_ *is compact on* $B_{\text{log}}^{\varphi }$ *if and only if ψC*_*ϕ*_ *is bounded on* $B_{\text{log}}^{\varphi }$,

$$\begin{array}{c} \underset{\left|\phi \left(z\right)\right|\to 1^{-}}{\text{lim}}\frac{|\psi^{\prime }\left(z\right)|\left(2+\text{log}\;\text{log}\frac{2}{1-|\phi (z)|}\right)}{\varphi^{-1}\left(\frac{1}{{\left(1-|z\right|}^{2})\text{log}\frac{2}{1-|z|}}\right)}=0 \end{array}$$ (4.1)

*and*

$$\begin{array}{c} \underset{\left|\phi \left(z\right)\right|\to 1^{-}}{\text{lim}}\frac{|\psi \left(z\right)\phi^{\prime }\left(z\right)|\varphi^{-1}\left(\frac{1}{(1-|\phi \left(z\right){|}^{2}\text{log}\frac{2}{1-|\phi (z)|}}\right)}{\varphi^{-1}\left(\frac{1}{{\left(1-|z\right|}^{2})\text{log}\frac{2}{1-|z|}}\right)}=0. \end{array}$$ (4.2)

*Proof*. Suppose that the weighted composition operator *ψC*_*ϕ*_ is bounded on $B_{\text{log}}^{\varphi }$ and (4.1) (4.2) hold. Note that *L*_1_ < ∞ and *L*_2_ < ∞ defined in the proof of Theorem 3.3 (see, (3.2) and (3.3), respectively) by the boundedness of *ψC*_*ϕ*_. For every *ϵ* > 0, there exists an 0 < *r* < 1 such that for |*ϕ*(*z*)| > *r*,

$$\frac{|\psi^{\prime }\left(z\right)|\left(2+\text{log}\;\text{log}\frac{2}{1-|\phi (z)|}\right)}{\varphi^{-1}\left(\frac{1}{{\left(1-|z\right|}^{2})\text{log}\frac{2}{1-|z|}}\right)}<\frac{\epsilon }{2}$$

and

$$\frac{|\psi \left(z\right)\phi^{\prime }\left(z\right)|\varphi^{-1}\left(\frac{1}{{\left(1-|\phi \left(z\right)\right|}^{2})\text{log}\frac{2}{1-{\left|\phi \left(z\right)\right|}^{2}}}\right)}{{\left(1-|\phi \left(z\right)\right|}^{2})\varphi^{-1}\left(\frac{1}{{\left(1-|z\right|}^{2})\text{log}\frac{2}{1-|z|}}\right)}<\frac{\epsilon }{2}$$

hold. For a chosen sequence ${\left\{f_{n}\right\}}_{n}\subset B_{\text{log}}^{\varphi }$ that satisfy $\underset{n\in N}{\text{sup}}{\left\|f_{n}\right\|}_{B_{\text{log}}^{\varphi }}\leq K$ and {*f*_*n*_} converges to zero uniformly on any compact subsets of the unit disk as *n* → ∞, where *K* is a fixed constant. It is sufficient to show that $\underset{n\to \infty }{\text{lim}}{\left\|\psi C_{\phi }f_{n}\right\|}_{B_{\text{log}}^{\phi }}=0$ by the compactness of *ψC*_*ϕ*_. Note that $\underset{n\to \infty }{\text{lim}}f_{n}\left(0\right)=0$ and $\left\{f_{n}^{\prime }\right\}$ converges to zero uniformly on any compact subsets of the unit disk. It follows by Proposition 2.3 that

$$\begin{array}{cc} & \|\psi C_{\phi }f_{n}{\|}_{B_{\text{log}}^{\phi }}=\left|\psi \left(0\right)f_{n}\left(\phi \left(0\right)\right)\right|+\underset{z\in D}{\text{sup}}\frac{|{\left(\psi C_{\phi }f_{n}\right)}^{\prime }\left(z\right)|}{\varphi^{-1}\left(\frac{1}{{\left(1-|z\right|}^{2})\text{log}\frac{2}{1-|z|}}\right)}\\ & =|\psi \left(0\right)f_{n}\left(\phi \left(0\right)\right)|+\underset{z\in D}{\text{sup}}\frac{|f_{n}^{\prime }\left(\phi \left(z\right)\right)\phi^{\prime }\left(z\right)\psi \left(z\right)+f_{n}\left(\phi \left(z\right)\right)\psi^{\prime }\left(z\right)|}{\varphi^{-1}\left(\frac{1}{{\left(1-|z\right|}^{2})\text{log}\frac{2}{1-|z|}}\right)}\\ & \leq |\psi \left(0\right)f_{n}\left(\phi \left(0\right)\right)|+\underset{\left\{z\in D:|\phi (z)|\leq r\right\}}{\text{sup}}\frac{|f_{n}^{\prime }\left(\phi \left(z\right)\right)\phi^{\prime }\left(z\right)\psi \left(z\right)+f_{n}\left(\phi \left(z\right)\right)\psi^{\prime }\left(z\right)|}{\varphi^{-1}\left(\frac{1}{{\left(1-|z\right|}^{2})\text{log}\frac{2}{1-|z|}}\right)}\\ & \;+\underset{\left\{z\in D:|\phi (z)|>r\right\}}{\text{sup}}\frac{|f_{n}^{\prime }\left(\phi \left(z\right)\right)\phi^{\prime }\left(z\right)\psi \left(z\right)+f_{n}\left(\phi \left(z\right)\right)\psi^{\prime }\left(z\right)|}{\varphi^{-1}\left(\frac{1}{{\left(1-|z\right|}^{2})\text{log}\frac{2}{1-|z|}}\right)} \end{array}$$

$$\begin{array}{cc} & \leq |\psi \left(0\right)f_{n}\left(\phi \left(0\right)\right)|+L_{1}\underset{\left\{w\in D:|w|\leq r\right\}}{\text{sup}}|f_{n}\left(w\right)|+L_{2}\underset{\left\{w\in D:|w|\leq r\right\}}{\text{sup}}\left|f_{n}^{\prime }\left(w\right)\right|\\ & \;+\underset{\left\{z\in D:|\phi (z)|>r\right\}}{\text{sup}}\frac{\varphi^{-1}\left(\frac{1}{{\left(1-|\phi \left(z\right)\right|}^{2})\text{log}\frac{2}{1-|\phi (z)|}}\right){\left\|f_{n}\right\|}_{B_{\text{log}}^{\varphi }}}{\varphi^{-1}\left(\frac{1}{{\left(1-|z\right|}^{2})\text{log}\frac{2}{1-|z|}}\right)}\left|\phi^{\prime }\left(z\right)\psi \left(z\right)\right|\\ & \;+\underset{\left\{z\in D:|\phi (z)|>r\right\}}{\text{sup}}\frac{\left(2+\text{log}\;\text{log}\frac{2}{1-|\phi (z)|}\right){\left\|f_{n}\right\|}_{B_{\text{log}}^{\varphi }}}{\varphi^{-1}\left(\frac{1}{{\left(1-|z\right|}^{2})\text{log}\frac{2}{1-|z|}}\right)}\left|\psi^{\prime }\left(z\right)\right|≲\epsilon . \end{array}$$

Then we conclude that *ψC*_*ϕ*_ is compact on $B_{\text{log}}^{\varphi }$ by the arbitrariness of *ϵ* > 0.

Conversely, suppose that *ψC*_*ϕ*_ is compact on $B_{\text{log}}^{\varphi }$ and hence *ψC*_*ϕ*_ is bounded on $B_{\text{log}}^{\varphi }$. We prove (4.1) and (4.2) hold as follows. Let {*z*_*n*,log_}_*n*_ be a sequence in the unit disk satisfying $\underset{n\to \infty }{\text{lim}}\left|\phi \left(z_{n,\text{log}}\right)\right|=1.$ If such sequence does not exist, then the proof is completed.

On the one hand, for each z∈D, we consider the function ${\tilde{p}}_{\phi (z_{n,\text{log}})}\left(z\right)$, where ${\tilde{p}}_{a}$ is constructed in Lemma 4.1. Then we have ${\tilde{p}}_{\phi (z_{n,\text{log}})}^{\prime }\left(\phi \left(z_{n,\text{log}}\right)\right)=0$ and

$${\tilde{p}}_{\phi (z_{n,\text{log}})}\left(\phi \left(z_{n,\text{log}}\right)\right)=\text{log}\;\text{log}\frac{4}{1-|\phi \left(z_{n,\text{log}}\right){|}^{2}}.$$

Note that ${\left\{{\tilde{p}}_{\phi (z_{n,\text{log}})}\right\}}_{n}$ is bounded uniformly in $B_{\text{log}}^{\varphi }$ and uniformly converges to zero on any compact subset of the unit disk as *n* → ∞ by (1.1). Thus we have $\underset{n\to \infty }{\text{lim}}\;{\left\|\psi C_{\phi }{\tilde{p}}_{\phi (z_{n,\text{log}})}\right\|}_{B_{\text{log}}^{\phi }}=0.$ It follows that

$$\underset{n\to \infty }{\text{lim}}\frac{|\psi^{\prime }\left(z_{n,\text{log}}\right)|}{\varphi^{-1}\left(\frac{1}{\left(1-\right|z_{n,\text{log}}{|}^{2})\text{log}\frac{2}{1-|z_{n,\text{log}}|}}\right)}\leq \underset{n\to \infty }{\text{lim}}\frac{|\psi^{\prime }\left(z_{n,\text{log}}\right)|\text{log}\;\text{log}\frac{2}{1-|\phi (z_{n,\text{log}})|}}{\varphi^{-1}\left(\frac{1}{\left(1-\right|z_{n,\text{log}}{|}^{2})\text{log}\frac{2}{1-|z_{n,\text{log}}|}}\right)}=0$$

and hence

$$\underset{\left|\phi \left(z\right)\right|\to 1^{-}}{\text{lim}}\frac{|\psi^{\prime }\left(z\right)|\left(2+\text{log}\;\text{log}\frac{2}{1-|\phi (z)|}\right)}{\varphi^{-1}\left(\frac{1}{{\left(1-|z\right|}^{2})\text{log}\frac{2}{1-|z|}}\right)}=0.$$

On the other hand, we consider the function $q_{\phi \left(z_{n},\text{log}\right)}(z)$, where *q*_*a*_ is constructed in Lemma 3.2. Then we have $q_{\phi \left(z_{n},\text{log}\right)}(\phi (z_{n,\text{log}}))=0$ and

$$q_{\phi (z_{n,\text{log}})}^{\prime }\left(\phi \left(z_{n,\text{log}}\right)\right)=\varphi^{-1}\left(\frac{1}{(1-|\phi \left(z_{n,\text{log}}\right){|}^{2})\text{log}\frac{2}{1-|\phi (z_{n,\text{log}})|}}\right).$$

Note that ${\left\{q_{\phi (z_{n},\text{log})}\right\}}_{n}$ is bounded uniformly in $B_{\text{log}}^{\varphi }$ and uniformly converges to zero on any compact subset of the unit disk as *n* → ∞ since

$$\begin{array}{cc} & \varphi^{-1}\left(\frac{1}{(1-|\phi \left(z_{n,\text{log}}\right){|}^{2})\text{log}\frac{2}{1-|\phi (z_{n,\text{log}})|}}\right)\frac{\left(1-|\phi \left(z_{n,\text{log}}\right){|}^{2}\right)}{\left|\phi (z_{n,\text{log}})\right|}\text{log}\left(1-\bar{\phi (z_{n,\text{log}})}z\right)\\ & \leq \varphi^{-1}\left(1\right)\frac{1}{\text{log}\frac{2}{1-|\phi (z_{n,\text{log}})|}}\frac{1}{\left|\phi (z_{n,\text{log}})\right|}\text{log}\left(1-\bar{\phi (z_{n,\text{log}})}z\right) \end{array}$$

Thus we have $\underset{n\to \infty }{\text{lim}}{\left\|\psi C_{\phi }q_{\phi (z_{n,\text{log}})}\right\|}_{B_{\text{log}}^{\phi }}=0.$ It follows that

$$\begin{array}{cc} & \underset{n\to \infty }{\text{lim}}\frac{|\psi \left(z_{n,\text{log}}\right)\phi^{\prime }\left(z_{n,\text{log}}\right)|\varphi^{-1}\left(\frac{1}{(1-|\phi \left(z_{n,\text{log}}\right){|}^{2})\text{log}\frac{2}{1-|\phi (z_{n,\text{log}})|}}\right)}{\varphi^{-1}\left(\frac{1}{\left(1-\right|z_{n,\text{log}}{|}^{2})\text{log}\frac{2}{1-|z_{n,\text{log}}|}}\right)}\\ & \leq \underset{n\to \infty }{\text{lim}}{\left\|\psi C_{\phi }q_{\phi (z_{n,\text{log}})}\right\|}_{B_{\text{log}}^{\varphi }}+\underset{n\to \infty }{\text{lim}}\frac{|\psi^{\prime }\left(z_{n,\text{log}}\right)|\left(2+\text{log}\;\text{log}\frac{2}{1-|\phi (z_{n,\text{log}})|}\right)}{\varphi^{-1}\left(\frac{1}{\left(1-\right|z_{n,\text{log}}{|}^{2})\text{log}\frac{2}{1-|z_{n,\text{log}}|}}\right)}=0 \end{array}$$

By the same arguments shown in the proof of the boundedness of *ψC*_*ϕ*_ on $B_{\text{log}}^{\varphi }$, we conclude that

$$\underset{\left|\phi \left(z\right)\right|\to 1^{-}}{\text{lim}}\frac{|\psi \left(z\right)\phi^{\prime }\left(z\right)|\varphi^{-1}\left(\frac{1}{(1-|\phi \left(z\right){|}^{2}\text{log}\frac{2}{1-|\phi (z)|}}\right)}{\varphi^{-1}\left(\frac{1}{{\left(1-|z\right|}^{2})\text{log}\frac{2}{1-|z|}}\right)}=0.$$

Combining what we have observed above, we complete the proof.

## 5 Boundedness and compactness of weighted composition operators on $B_{\mu_{\text{log}}}$

Recall that it is proved in Proposition 2.3 that the logarithmic Bloch-Orlicz space is isometrically equal to a *μ*_log_–Bloch space, where

$$\mu_{\text{log}}\left(z\right)=\frac{1}{\varphi^{-1}\left(\frac{1}{{\left(1-|z\right|}^{2})\text{log}\frac{2}{1-|z|}}\right)}.$$

Hence, the boundedness and compactness of weighted composition operators on the *μ*_log_–Bloch space $B_{\mu_{\text{log}}}$ could be naturally given.

**Theorem 5.1** *For* φ∈U , *the weighted composition operator ψC*_*ϕ*_ *is bounded on* $B_{\mu_{\text{log}}}$ *if and only if*

$$\begin{array}{c} M_{1}≔\underset{z\in D}{\text{sup}}\;\mu_{\text{log}}\left(z\right)\left|\psi^{\prime }\left(z\right)\right|\left(2+\text{log}\;\text{log}\frac{2}{1-|z|}\right)<\infty \end{array}$$

*and*

$$\begin{array}{c} M_{2}≔\underset{z\in D}{\text{sup}}\frac{\mu_{\text{log}}\left(z\right)\left|\psi \left(z\right)\phi^{\prime }\left(z\right)\right|}{\mu_{\text{log}}\left(\phi \left(z\right)\right)}<\infty \end{array}$$

*hold*.

**Theorem 5.2** *For* φ∈U , *the weighted composition operator ψC*_*ϕ*_ *is compact on* $B_{\mu_{\text{log}}}$ *if and only if ψC*_*ϕ*_ *is bounded on* $B_{\mu_{\text{log}}}$,

$$\begin{array}{c} \underset{\left|\phi \left(z\right)\right|\to 1^{-}}{\text{lim}}\mu_{\text{log}}\left(z\right)\left|\psi^{\prime }\left(z\right)\right|\left(2+\text{log}\;\text{log}\frac{2}{1-|z|}\right)=0 \end{array}$$

*and*

$$\begin{array}{c} \underset{\left|\phi \left(z\right)\right|\to 1^{-}}{\text{lim}}\frac{\mu_{\text{log}}\left(z\right)\left|\psi \left(z\right)\phi^{\prime }\left(z\right)\right|}{\mu_{\text{log}}\left(\phi \left(z\right)\right)}=0. \end{array}$$

## Supporting information

S1 File (PDF)

S2 File (DOCX)

## References

[1] Cowen C. C., Maccluer B. D., Composition operators on spaces of analytic functions, CRC Press, 1995.

[2] Bai H. B., Jiang Z. J., Generalized weighted composition operators from Zygmund spaces to Bloch-Orlicz type spaces, Appl. Math. Comput. 273 (2016) 89–97.

[3] Giménez J., Malavé R., Ramos-Fernández J., Composition operators on *μ*–Bloch type spaces, Rend. Circ. Mat. Palermo 59 (2010) 107–119. doi: 10.1007/s12215-010-0007-1

[4] Liang Y. X., Integral-type operators from *F*(*p*, *q*, *s*) space to *α*-Bloch-Orlicz and *β*-Zygmund-Orlicz spaces, Complex Anal. Oper. Theory 10:8 (2016) 1–26.

[5] Xiao J., Composition operators associated with Bloch-type spaces, Complex Var. Theor. Appl. 46 (2001) 109–121.

[6] Yoneda R., The composition operators on weighted Bloch space, Arch. Math. (Basel) 78 (2002) 310–317. doi: 10.1007/s00013-002-8252-y

[7] Zhou H., Boundedness and compactness of specific weighted composition operators on the Zygmund-Orlicz Space, Journal of Function Spaces, 2022. doi: 10.1155/2022/5042795

[8] Charpentier S., Composition operators on Hardy-Orlicz spaces on the ball, Integral Equations Operator Theory 70 (2011) 429–450. doi: 10.1007/s00020-011-1870-7

[9] Jiang Z., Cao G., Composition operator on Bergman-Orlicz space, J. Inequal. Appl. 1 (2009) 1–15.

[10] Li D., Compact composition operators on Hardy-Orlicz and Bergman-Orlicz spaces, RACSAM 105 (2011) 247–260.

[11] Lefèvre P., Li D., Queffélec H., Rodríguez-Piazza L., Composition operators on Hardy-Orlicz spaces, American Mathematical Society, 207 (2010).

[12] Lefèvre P., Li D., Queffélec H., Rodríguez-Piazza L., Compact composition operators on Bergman-Orlicz spaces, Trans. Amer. Math. Soc. 365 (2013) 3943–3970. doi: 10.1090/S0002-9947-2013-05922-3

[13] Lefèvre P., Li D., Queffélec H., Rodríguez-Piazza L., Compact composition operators on *H*^2^ and Hardy-Orlicz spaces, J. Math. Anal. Appl. 354 (2009) 360–371. doi: 10.1016/j.jmaa.2009.01.004

[14] Sharma A. K., Sharma S.D., Composition operators on weighted Bergman-Orlicz spaces, Bull. Austral. Math. Soc. 75 (2007) 273–287. doi: 10.1017/S0004972700039204

[15] Fernández J. C. R., Composition operators on Bloch-Orlicz type spaces, Appl. Math. Comput. 217 (2010) 3392–3402.

[16] Liang Y. X., Volterra-type operators from weighted Bergman-Orlicz space to *β*–Zygmund-Orlicz and *γ*–Bloch-Orlicz spaces, Monatsh. Math. 182 (2017) 877–897. doi: 10.1007/s00605-016-0986-x

[17] Ye S., Multipliers and cyclic vectors on the weighted Bloch space, Math. J. Okayama Univ. 48 (2006), 135–143.

