# Supplementary material for: Weighted composition operators on the logarithmic Bloch-Orlicz space
Source: PLoS One. 2024 May 28;19(5):e0303336. doi: 10.1371/journal.pone.0303336 (PMC11132511; doi:10.1371/journal.pone.0303336)
Supplement: S1 File — (PDF) [file pone.0303336.s001.pdf]

Weighted Composition Operators on the Logarithmic Bloch-Orlicz Space

Hang Zhou[1*],

**1** Department of Information Technology and Engineering, Guangzhou College of Commerce, Guangzhou, G. D. 511363, P.R. China

**Keywords.** weighted composition operators, boundedness, compactness, logarithmic Bloch-Orlicz space
**subjclass[2010].** Primary: 47B38; Secondary: 47B33, 32H02.
This work was supported by the Department of Education of Guangdong Province (Grant Nos. 2022KQNCX121) and there was no additional external funding received for this study.
* corresponding author

## Abstract

The boundedness and compactness of weighted composition operators on the logarithmic Bloch-Orlicz space $\mathcal{B}^{\varphi}_{\log}$ are investigated in this paper.

## 1 Introduction

Suppose that $S(\mathbb{D})$ is the set of all analytic self-maps of the unit disk $\mathbb{D}$ (the analytic maps from $\mathbb{D}$ to itself). Let $H(\mathbb{D})$ be, as usual, the collection of all analytic functions on $\mathbb{D}$. The composition operator, induced by an analytic self-map of the unit disk $\varphi$, could be defined as

$$C_\phi f = f \circ \phi, f \in H(\mathbb{D}).$$

For $\psi \in H(\mathbb{D})$, the weighted composition operator could be defined as

$$\psi C_\phi f = \psi \cdot f \circ \phi, f \in H(\mathbb{D}).$$

The study of composition operators and weighted composition operators has been established for over 6 decades. Many mathematicians aimed at the research of (weighted) composition operators on spaces of analytic functions on $\mathbb{D}$ or on some high-dimension domains (e.g., the unit ball of $\mathbb{C}^n$, the unit polydisk of $\mathbb{C}^n$). We refer to reference [3] for studying the history of composition operators acting on different spaces of analytic functions.

As one of the classical space of analytic functions on $\mathbb{D}$, the Bloch space, is defined as

$$\mathcal{B} = \{f \in H(\mathbb{D}) : \|f\|_{\mathcal{B}} = \sup_{z\in\mathbb{D}} (1 - |z|^2)|f'(z)| < \infty\}.$$

Importantly, $\mathcal{B}$ is maximal among all Möbius-invariant spaces of analytic functions and $\mathcal{B}$ turns to a complete normed linear space endowed with the norm

$$\|f\|_1 = |f(0)| + \|f\|_{\mathcal{B}}.$$

The logarithmic Bloch space $\mathcal{B}_{log}$ is defined as

$$\mathcal{B}_{log} = \{f \in H(\mathbb{D}) : \|f\|_{\mathcal{B}_{log}} = \sup_{z\in\mathbb{D}}(1-|z|^2)\log\frac{2}{1-|z|}|f'(z)| < \infty\}.$$

It is a Banach space endowed with the norm $\|f\|_{log} = |f(0)| + \|f\|_{\mathcal{B}_{log}}$.

The $\mu-$Bloch space $\mathcal{B}_\mu$ is defined as

$$\mathcal{B}_\mu = \{f \in H(\mathbb{D}) : \|f\|_{\mathcal{B}_\mu} = \sup_{z\in\mathbb{D}}\mu(z)|f'(z)| < \infty\},$$

where the weight function $\mu(z)$ is a positive continuous function in $\mathbb{D}$. Then $\mathcal{B}_\mu$ is a Banach space endowed with the norm $\|f\|_\mu = |f(0)| + \|f\|_{\mathcal{B}_\mu}$.

Recall that a Young's function $\varphi : [0,\infty) \to [0,\infty)$ is a strictly increasing convex function such that $\varphi(0) = 0$ and $\lim_{x\to\infty}\varphi(x) = \infty$.

In the recent years, the boundedness and compactness of composition operators among different Bloch(-type) spaces were studied (see, e.g., [5, 15, 16] and the references therein). Moreover, as the study of Hardy-Orlicz space and Bergman-Orlicz space (see, e.g., [2, 6, 9–12, 14]), the Bloch-Orlicz space $\mathcal{B}^\varphi$ was defined as a generalization of $\mathcal{B}$.

It was firstly defined in [4],

$$\mathcal{B}^\varphi = \{f \in H(\mathbb{D}) : \sup_{z\in\mathbb{D}}(1-|z|^2)\varphi(\lambda|f'(z)|) < \infty\}$$

for some $\lambda > 0$ depending of $f$, where $\varphi$ is the Young's function. Basic properties of the convex function imply

$$\varphi(st) \le s\varphi(t), s < 1, t > 0,$$
$$\varphi(st) \ge s\varphi(t), s > 1, t > 0$$

and

$$\varphi^{-1}(st) \le s\varphi^{-1}(t), s > 1, t > 0. \tag{1.1}$$

We can assume without loss of generality that $\varphi^{-1}$ is differentiable (as the authors did in [4]). Suppose that

$$S_\varphi(f) := \sup_{z\in\mathbb{D}}(1-|z|^2)\varphi(|f(z)|),$$

then

$$\|f\|_\varphi = \inf\{k > 0 : S_\varphi(\frac{f'}{k}) \le 1\}$$

defines a semi-norm for $\mathcal{B}^\varphi$. In this way, $\mathcal{B}^\varphi$ is a Banach space endowed with the norm

$$\|f\|_{\mathcal{B}^\varphi} := |f(0)| + \|f\|_\varphi.$$

In this paper, we generalize the Bloch-Orlicz space to the logarithmic Bloch-Orlicz space. Note that it is a generalization of the logarithmic Bloch space $\mathcal{B}_{log}$. The logarithmic Bloch-Orlicz space $\mathcal{B}^\varphi_{log}$ is defined as follows,

$$\mathcal{B}^\varphi_{\log} = \{f \in H(\mathbb{D}) : \sup_{z\in\mathbb{D}}(1-|z|^2)\log\frac{2}{1-|z|}\varphi(\lambda|f'(z)|) < \infty\}$$

for some $\lambda > 0$ depending of $f$, where $\varphi$ is the Young's function and $\varphi^{-1}$ is differentiable. Moreover,

$$\|f\|_{\varphi,\log} = \inf\{k > 0 : S_{\varphi,\log}(\frac{f'}{k}) \le 1\}$$

defines a semi-norm for $\mathcal{B}^{\varphi}_{\log}$, where

$$S_{\varphi,\log}(f) := \sup_{z\in\mathbb{D}}(1-|z|^2)\log\frac{2}{1-|z|}\varphi(|f(z)|).$$

Thus, $\mathcal{B}^{\varphi}_{\log}$ becomes a Banach space endowed with the norm

$$\|f\|_{\mathcal{B}^{\varphi}_{\log}} := |f(0)| + \|f\|_{\varphi,\log}.$$

This paper is organized as follows: in Section 2 we recall some basic facts on the logarithmic Bloch-Orlicz space. In Section 3 we investigate the boundedness of weighted composition operators on the logarithmic Bloch-Orlicz space. Moreover, in Section 4 we investigate the compactness of weighted composition operators on the logarithmic Bloch-Orlicz space.

We say that $\varphi \in \mathcal{U}$ if there exists a constant $M_\varphi > 1$ such that

$$\varphi(st) \le M_\varphi s\varphi(t), s > 1, t > 0,$$

which also implies that

$$s\varphi^{-1}(t) \le \varphi^{-1}(st) \le M_\varphi s\varphi^{-1}(t), s < 1, t > 0$$

and

$$s\varphi^{-1}(t) \ge \varphi^{-1}(st) \ge \frac{1}{M_\varphi}s\varphi^{-1}(t), s > 1, t > 0.$$

## 2 Preliminaries

In this section, we present several basic conclusions for the study of $\mathcal{B}^{\varphi}_{\log}$.

**Proposition 2.1** *[7]*

$$S_{\varphi,\alpha}(\frac{f'}{\|f\|_{\mathcal{B}^{\varphi}_{\log}}}) \le S_{\varphi,\alpha}(\frac{f'}{\|f\|_{\varphi,\log}}) \le 1$$

*holds for each* $f \in \mathcal{B}^{\varphi}_{\log}$.

*Proof.* The proof is similar with Lemma 2 in [4].

For each $f \in \mathcal{B}^{\varphi}_{\log} \setminus \{0\}$, a decreasing sequence $\{\lambda_{n,\log}\}_n$ of positive numbers can be chosen, satisfying $\lim_{n\to\infty}\lambda_{n,\log} = \|f\|_{\varphi,\log}$ and $S_{\varphi,\log}(\frac{f'}{\lambda_{n,\log}}) \le 1$. For any positive integer $n \in \mathbb{N}$, let $S_n := S_{\varphi,\log}(\frac{f'}{\lambda_{n,\log}})$. Observe that $\{S_n\}$ is increasing and bounded and hence there exists a real number $S' \in \mathbb{R}$ satisfying $\lim_{n\to\infty} S_n = S'$. It follows that $S' = \sup_{n\in\mathbb{N}}\{S_n\} \le 1$. Observe that

$$S_n = S_{\varphi,\log}(\frac{f'}{\lambda_{n,\log}}) \le S_{\varphi,\log}(\frac{f'}{\|f\|_{\varphi,\log}}) := S.$$

Thus we have $S' \le S$. Moreover,

$$(1-|z|^2)\log\frac{2}{1-|z|}\varphi(\frac{|f'(z)|}{\lambda_{n,\log}}) \le S_{\varphi,\log}(\frac{f'}{\lambda_{n,\log}}) \le S' \le 1$$

holds for each $z \in \mathbb{D}$ and $n \in \mathbb{N}$. Taking limit as $n \to \infty$, the above inequality becomes

$$(1-|z|^2)\log\frac{2}{1-|z|}\varphi(\frac{|f'(z)|}{\|f\|_{\varphi,\log}}) \le S_{\varphi,\log}(\frac{f'}{\lambda_{n,\log}}) \le S' \le 1$$

for each $z \in \mathbb{D}$, which is equivalent to say that

$$S = \sup_{z\in\mathbb{D}}(1-|z|^2)\log\frac{2}{1-|z|}\varphi(\frac{|f'(z)|}{\|f\|_{\varphi,\log}}) \leq 1.$$

This completes the proof. □

**Remark 2.2** *The inequality*

$$|f(z)| \leq M_\varphi \varphi^{-1}(1)(2+\log\log\frac{2}{1-|z|})\|f\|_{\mathcal{B}^\varphi_{\log}} \tag{2.1}$$

*holds for all* $f \in \mathcal{B}^\varphi_{\log}$ *and* $z \in \mathbb{D}$ *by Lemma 2.1, where* $M_\varphi > 1$ *is a constant only dependent of* $\varphi$*. In fact, a simple estimation shows that*

$$\begin{aligned}|f(z)-f(tz)| = |z\int_t^1 f'(zt)dt| &\leq \|f\|_{\varphi,\log}\int_t^1 \varphi^{-1}(\frac{1}{(1-|zt|^2)\log\frac{2}{1-|zt|}})dt \\ &\leq M_\varphi\varphi^{-1}(1)\|f\|_{\varphi,\log}\int_t^1 \frac{1}{1-|zt|^2}\log\frac{2}{1-|zt|}dt \\ &\leq M_\varphi\varphi^{-1}(1)\log\frac{\log\frac{2}{1-|z|}}{\log\frac{2}{1-|tz|}}\|f\|_{\varphi,\log},\end{aligned}$$

*where more details can be found in [17]. The inequality above also implies that the evaluation functional is continuous on* $\mathcal{B}^\varphi_{\log}$*, where* $z \in \mathbb{D}$ *is fixed.*

The proposition below shows that the logarithmic Bloch-Orlicz space is isometrically equal to a $\mu-$Bloch space.

**Proposition 2.3** *The logarithmic Bloch-Orlicz space is isometrically equal to a* $\mu_{\log}-$*Bloch space, where*

$$\mu_{\log}(z) = \frac{1}{\varphi^{-1}(\frac{1}{(1-|z|^2)\log\frac{2}{1-|z|}})}.$$

*In other words,*

$$\|f\|_{\mathcal{B}^\varphi_{\log}} = |f(0)| + \sup_{z\in\mathbb{D}}\mu_{\log}(z)|f'(z)|$$

*holds for each* $f \in \mathcal{B}^\varphi_{\log}$.

*Proof.* Deducted by Proposition 2.1, for each $f \in \mathcal{B}^\varphi_{\log}$ and $z \in \mathbb{D}$,

$$(1-|z|^2)\log\frac{2}{1-|z|}\varphi(\frac{|f'(z)|}{\|f\|_{\varphi,\log}}) \leq 1,$$

which implies that $\mu_{\log}(z)|f'(z)| \leq \|f\|_{\varphi,\log}$ holds for all $z \in \mathbb{D}$. Therefore, $\mathcal{B}^\varphi_{\log} \subset \mathcal{B}_{\mu_{\log}}$ with $\|f\|_{\mu_{\log}} \leq \|f\|_{\varphi,\log}$. Conversely, for each $f \in \mathcal{B}_{\mu_{\log}}$, $\mu_{\log}(z)|f'(z)| \leq \|f\|_{\mu_{\log}}$ holds for all $z \in \mathbb{D}$, which is equivalent with

$$\frac{1}{\varphi^{-1}(\frac{1}{(1-|z|^2)\log\frac{2}{1-|z|}})}|f'(z)| \leq \|f\|_{\mu_{\log}}.$$

It follow that $S_{\varphi,\log}(\frac{f'}{\|f\|_{\mu_{\log}}}) \leq 1$. Therefore, we obtain that $\mathcal{B}_{\mu_{\log}} \subset \mathcal{B}^\varphi_{\log}$ with $\|f\|_{\varphi,\log} \leq \|f\|_{\mu_{\log}}$. Combining what we have observed above, we complete the proof. □

**Corollary 2.4** *The equivalent condition*

$$S_{\varphi,\log}(f') \leq 1 \Leftrightarrow \|f\|_{\varphi,\log} \leq 1$$

*holds for each* $f \in \mathcal{B}^{\varphi}_{\log}$.

*Proof.* The sufficiency part is obvious. The necessity is deducted by Proposition 2.1 and the estimation $S_{\varphi,\log}(f') \leq S_{\varphi,\log}(\frac{f'}{\|f\|_{\varphi,\log}}) \leq 1$. □

For two real numbers $A$ and $B$, we say $A \lesssim B$ if there exists a constant $C \neq 0$ such that $A \leq CB$, by which the complexity of all constants appearing is simplified.

# 3 Boundedness of Weighted Composition Operator on $\mathcal{B}^{\varphi}_{\log}$

In this section we investigate the boundedness of weighted composition operators on the logarithmic Bloch-Orlicz space under the condition $\varphi \in \mathcal{U}$(this is an unexpected hypothesis). However, for those Young's funtion $\varphi \notin \mathcal{U}$, the boundedness of weighted composition operators on the logarithmic Bloch-Orlicz space remains to be an open question.

The first lemma contains some trivial but complicated calculations, which will be used in the proof in this section.

**Lemma 3.1** *Let*

$$g_t(z) = \frac{(1-|z|)\log\frac{2}{1-|z|}}{(1-|tz|)\log\frac{2}{1-|tz|}},$$

*where* $t \in [0,1]$ *and* $z \in \mathbb{D}$*, then* $|g_t(z)| < 2$.

Basic properties of the auxiliary functions entailed to the proof of the boundedness of weighted composition operator $\psi C_\phi$ are described in the next lemma.

**Lemma 3.2** *For* $a \in \mathbb{D}$*,* $n \in \mathbb{N}^+$ *and* $\varphi \in \mathcal{U}$*, suppose that*

$$p_a(z) = 2\log\log\frac{4}{1-\overline{\phi(a)}z} - \frac{1}{\log\log\frac{4}{1-|\phi(a)|^2}}(\log\log\frac{4}{1-\overline{\phi(a)}z})^2$$

*and*

$$q_a(z) = \varphi^{-1}(\frac{1}{(1-|\phi(a)|^2)\log\frac{2}{1-|\phi(a)|}})\frac{-(1-|\phi(a)|^2)}{\overline{\phi(a)}}\log(1-\overline{\phi(a)}z),$$

*where* $z \in \mathbb{D}$*. Then the auxiliary functions* $p_a$ *and* $q_a$ *have the properties as follows:*

*(i) the auxiliary function* $p_a$ *belongs to the logarithmic Bloch-Orlicz space* $\mathcal{B}^{\varphi}_{\log}$ *with* $\sup\limits_{a\in\mathbb{D}} \|p_a\|_{\mathcal{B}^{\varphi}_{\log}} \lesssim 1$.

*(ii) the auxiliary function* $q_a$ *belongs to the logarithmic Bloch-Orlicz space* $\mathcal{B}^{\varphi}_{\log}$ *with* $\sup\limits_{a\in\mathbb{D}} \|q_a\|_{\mathcal{B}^{\varphi}_{\log}} \lesssim 1$.

*Proof.* As easy calculation shows,

$$p'_a(z) = \frac{2\overline{\varphi(a)}}{(1-\overline{\varphi(a)}z)\log\frac{4}{1-\overline{\varphi(a)}z}} - 2\frac{\log\log\frac{4}{1-\overline{\varphi(a)}z}}{\log\log\frac{4}{1-|\varphi(a)|^2}}\frac{\overline{\varphi(a)}}{(1-\overline{\varphi(a)}z)\frac{4}{1-\overline{\varphi(a)}z}}$$

and

$$q'_a(z) = \varphi^{-1}(\frac{1}{(1-|\phi(a)|^2)\log\frac{2}{1-|\phi(a)|}})\frac{1-|\phi(a)|^2}{|1-\overline{\phi(a)}z|}$$

Observe that

$$\begin{aligned}
&\|q_a\|_{\varphi,\log} \\
&= \sup_{z\in\mathbb{D}} \frac{1}{\varphi^{-1}(\frac{1}{(1-|z|^2)\log\frac{2}{1-|z|}})}\varphi^{-1}(\frac{1}{(1-|\phi(a)|^2)\log\frac{2}{1-|\phi(a)|}})\frac{1-|\phi(a)|^2}{|1-\overline{\phi(a)}z|} \\
&\le M_\varphi \sup_{z\in\mathbb{D}} \frac{(1-|z|^2)\log\frac{2}{1-|z|}}{|1-\overline{\phi(a)}z|\log\frac{2}{|1-\overline{\phi(a)}z|}}\frac{\log\frac{2}{|1-\overline{\phi(a)}z|}}{\log\frac{2}{1-|\phi(a)|}}\frac{1-|\phi(a)|^2}{1-|\phi(a)|^2} \le 4M_\varphi.
\end{aligned}$$

Then we conclude that $\sup\limits_{a\in\mathbb{D}} S_{\varphi,\log}(q_a') \lesssim 1$. Further observe that, by Lemma 3.1

$$\begin{aligned}
&\sup_{z\in\mathbb{D}} \frac{1}{\varphi^{-1}(\frac{1}{(1-|z|^2)\log\frac{2}{1-|z|}})}\frac{2|\phi(a)|}{|1-\overline{\phi(a)}z|\log\frac{4}{|1-\overline{\phi(a)}z|}} \\
&\le 2\sup_{z\in\mathbb{D}} \frac{4C_\phi(1-|z|)\log\frac{2}{1-|z|}}{|1-\overline{\phi(a)}z|\log\frac{2}{|1-\overline{\phi(a)}z|}} \lesssim 2
\end{aligned}$$

and

$$\begin{aligned}
&\sup_{z\in\mathbb{D}} \frac{1}{\varphi^{-1}(\frac{1}{(1-|z|^2)\log\frac{2}{1-|z|}})}\frac{2|\phi(a)|}{|1-\overline{\phi(a)}z|\log\frac{4}{|1-\overline{\phi(a)}z|}}\frac{\log\log\frac{4}{|1-\overline{\varphi(a)}z|}}{\log\log\frac{4}{1-|\varphi(a)|^2}} \\
&\lesssim \sup_{z\in\mathbb{D}} \frac{(1-|z|)\log\frac{2}{1-|z|}}{|1-\overline{\phi(a)}z|\log\frac{2}{|1-\overline{\phi(a)}z|}} \lesssim 2.
\end{aligned}$$

Then we conclude that $\sup\limits_{a\in\mathbb{D}} S_{\varphi,\log}(p_a') \lesssim 2$. □

Though the approach we use in the proof of the boundedness is standard, it is not trivial since the collection $\mathcal{U}$ contains not only (almost) linear functions, it also contains the convex functions which line between the the line whose tangent is 1 and a line whose tangent is $M_\varphi$, a positive number depending on $\varphi$.

**Theorem 3.3** *For $\varphi \in \mathcal{U}$, the weighted composition operator $\psi C_\phi$ is bounded on $\mathcal{B}_{\log}^{\varphi}$ if and only if*

$$M_1 := \sup_{z\in\mathbb{D}} \frac{|\psi'(z)|(2+\log\log\frac{2}{1-|z|})}{\varphi^{-1}(\frac{1}{(1-|z|^2)\log\frac{2}{1-|z|}})} < \infty$$

*and*

$$M_2 := \sup_{z\in\mathbb{D}} \frac{|\psi(z)\phi'(z)|\varphi^{-1}(\frac{1}{(1-|\phi(z)|^2\log\frac{2}{1-|\phi(z)|}})}{\varphi^{-1}(\frac{1}{(1-|z|^2)\log\frac{2}{1-|z|}})} < \infty$$

*hold.*

*Proof.* Suppose that $M_1<\infty, M_2<\infty$. For each $f\in \mathcal{B}^{\varphi}_{\log}\setminus\{0\}$, observe that

$$\begin{aligned}
&S_{\varphi,\log}(\frac{(\psi C_\phi f)'}{C\|f\|_{\mathcal{B}^{\varphi}_{\log}}})\\
&\le \sup_{z\in\mathbb{D}}(1-|z|^2)\log\frac{2}{1-|z|}\varphi(\frac{|\psi'(z)f(\phi(z))|}{C\|f\|_{\mathcal{B}^{\varphi}_{\log}}}+\frac{|\psi(z)f'(\phi(z))\phi'(z)|}{C\|f\|_{\mathcal{B}^{\varphi}_{\log}}})\\
&\le \sup_{z\in\mathbb{D}}(1-|z|^2)\log\frac{2}{1-|z|}\varphi(\frac{|\psi'(z)|M_\varphi\varphi^{-1}(1)(2+\log\log\frac{2}{1-|z|})\|f\|_{\mathcal{B}^{\varphi}_{\log}}}{C\|f\|_{\mathcal{B}^{\varphi}_{\log}}}\\
&\quad+\frac{|\psi(z)\phi'(z)|\varphi^{-1}(\frac{1}{(1-|\phi(z)|^2)\log\frac{2}{1-|\phi(z)|}})\|f\|_{\mathcal{B}^{\varphi}_{\log}}}{C\|f\|_{\mathcal{B}^{\varphi}_{\log}}})\\
&\le \sup_{z\in\mathbb{D}}(1-|z|^2)\log\frac{2}{1-|z|}\varphi(\varphi^{-1}(\frac{1}{(1-|z|^2)\log\frac{2}{1-|z|}})(\frac{M_\varphi\varphi^{-1}(1)M_1}{C}+\frac{M_2}{C}))\\
&\le \sup_{z\in\mathbb{D}}(1-|z|^2)\log\frac{2}{1-|z|}\varphi(\varphi^{-1}(\frac{1}{(1-|z|^2)\log\frac{2}{1-|z|}}))=1,
\end{aligned}$$

where $C$ is chosen such that $M_\varphi\varphi^{-1}(1)M_1+M_2\le C$ and the second inequality is deducted by (2.1). Then we conclude that $\psi C_\phi$ is bounded on $\mathcal{B}^{\varphi}_{\log}$ by

$$\|\psi C_\phi f\|_{\varphi,\log}\le C\|f\|_{\mathcal{B}^{\varphi}_{\log}}$$

and the estimation (2.1) by taking $z=\phi(0)$.

Conversely, if $\psi C_\phi$ is bounded on $\mathcal{B}^{\varphi}_{\log}$, then there exists a constant $C\ge 0$ such that $\|\psi C_\phi f\|_{\mathcal{B}^{\varphi}_{\log}}\le C\|f\|_{\mathcal{B}^{\varphi}_{\log}}$ for each $0\ne f\in\mathcal{B}^{\varphi}_{\log}$. By Proposition 2.1 and the condition above,

$$S_{\varphi,\log}(\frac{(\psi C_\phi f)'}{C\|f\|_{\mathcal{B}^{\varphi}_{\log}}})\le S_{\varphi,\log}(\frac{(\psi C_\phi f)'}{\|\psi C_\phi f\|_{\mathcal{B}^{\varphi}_{\log}}})\le 1$$

holds for each $f\in\mathcal{B}^{\varphi}_{\log}$, which is equivalent with

$$\sup_{z\in\mathbb{D}}(1-|z|^2)\log\frac{2}{1-|z|}\varphi(\frac{|f'(\phi(z))\phi'(z)\psi(z)+f(\phi(z))\psi'(z)|}{C\|f\|_{\mathcal{B}^{\varphi}_{\log}}})\le 1. \tag{3.1}$$

Taking $f_0(z)=1\in\mathcal{B}^{\varphi}_{\log}$, employing (3.1) we obtain that

$$L_1:=\sup_{z\in\mathbb{D}}\frac{|\psi'(z)|}{\varphi^{-1}(\frac{1}{(1-|z|^2)\log\frac{2}{1-|z|}})}<\infty. \tag{3.2}$$

Further taking $\hat{f}_0=z\in\mathcal{B}^{\varphi}_{\log}$, employing (3.1) again we obtain that

$$\frac{|\phi'(z)\psi(z)+\phi(z)\psi'(z)|}{\varphi^{-1}(\frac{1}{(1-|z|^2)\log\frac{2}{1-|z|}})}\le C\|f\|_{\mathcal{B}^{\varphi}_{\log}}$$

holds for each $z\in\mathbb{D}$. It follows that

$$\begin{aligned}
L_2:&=\sup_{z\in\mathbb{D}}\frac{|\phi'(z)\psi(z)|}{\varphi^{-1}(\frac{1}{(1-|z|^2)\log\frac{2}{1-|z|}})}\\
&\le\sup_{z\in\mathbb{D}}\frac{|\phi(z)\psi'(z)|}{\varphi^{-1}(\frac{1}{(1-|z|^2)\log\frac{2}{1-|z|}})}\qquad+\sup_{z\in\mathbb{D}}\frac{|\phi'(z)\psi(z)+\phi(z)\psi'(z)|}{\varphi^{-1}(\frac{1}{(1-|z|^2)\log\frac{2}{1-|z|}})}<\infty.
\end{aligned} \tag{3.3}$$

On the one hand, for each $a, z \in \mathbb{D}$, suppose that

$$p_a(z) = 2\log\log\frac{4}{1-\overline{\phi(a)}z} - \frac{1}{\log\log\frac{4}{1-|\phi(a)|^2}}(\log\log\frac{4}{1-\overline{\phi(a)}z})^2.$$

Obviously, $p_a'(\phi(a)) = 0$ and

$$p_a(\phi(a)) = \log\log\frac{4}{1-|\phi(a)|^2}$$

By Lemma 3.2, we have that $p_a \in \mathcal{B}_{\log}^{\varphi}$. It follows by the boundedness of $\psi C_\phi$ that

$$\frac{|\psi'(a)|(\log\log\frac{4}{1-|\phi(a)|^2})}{\varphi^{-1}(\frac{1}{(1-|a|^2)\log\frac{2}{1-|a|}})} = \frac{|(\psi C_\phi p_a)'(a)|}{\varphi^{-1}(\frac{1}{(1-|a|^2)\log\frac{2}{1-|a|}})} < \infty$$

and hence by $L_1 < \infty$ we conclude that

$$M_1 = \sup_{z\in\mathbb{D}}\frac{|\psi'(z)|(2+\log\log\frac{2}{1-|\phi(z)|})}{\varphi^{-1}(\frac{1}{(1-|z|^2)\log\frac{2}{1-|z|}})} < \infty.$$

On the other hand, for each $a, z \in \mathbb{D}$, we define

$$q_a(z) = \varphi^{-1}(\frac{1}{(1-|a|^2)\log\frac{2}{1-|a|}})\frac{-(1-|a|^2)}{\overline{a}}\log(1-\overline{a}z),$$

By Lemma 3.2, $q_a \in \mathcal{B}_{\log}^{\varphi}$. Obviously,

$$q_a'(\phi(a)) = \varphi^{-1}(\frac{1}{(1-|\phi(a)|^2)\log\frac{2}{1-|\phi(a)|}}).$$

For each $a \in \mathbb{D}$, we have

$$\begin{aligned}
&\mu_{\log}(a)\varphi^{-1}(\frac{1}{(1-|\phi(a)|^2)\log\frac{2}{1-|\phi(a)|^2}})|\psi(a)\phi'(a)| \\
&\le \|\psi C_\phi q_a\|_{\mathcal{B}_{\log}^{\varphi}} + \mu_{\log}(a)|\psi'(a)q_a(\phi(a))| \\
&\le \|\psi C_\phi q_a\|_{\mathcal{B}_{\log}^{\varphi}} + \mu_{\log}(a)|\psi'(a)|(2+\log\log\frac{2}{1-|\phi(a)|})\|q_a\|_{\mathcal{B}_{\log}^{\varphi}} \\
&\le \|\psi C_\phi q_a\|_{\mathcal{B}_{\log}^{\varphi}} + M_1\|q_a\|_{\mathcal{B}_{\log}^{\varphi}} < \infty.
\end{aligned}$$

Then we conclude that

$$M_2 := \sup_{z\in\mathbb{D}}\frac{|\psi(z)\phi'(z)|\varphi^{-1}(\frac{1}{(1-|\phi(z)|^2\log\frac{2}{1-|\phi(z)|}})}{\varphi^{-1}(\frac{1}{(1-|z|^2)\log\frac{2}{1-|z|}})} < \infty.$$

Combining what we have observed above, we complete the proof. □

# 4 Compactness of Weighted Composition Operator on $\mathcal{B}_{\log}^{\varphi}$

In this section we investigate the compactness of weighted composition operators on the logarithmic Bloch-Orlicz space, where the approach we use in the proof within is standard (see, e.g., [7]).

The first lemma contains some trivial but complicated calculations, which will be used in the proof of the compactness of weighted composition operator on $\mathcal{B}_{\log}^{\varphi}$. Moreover, it can be proved in a similar way with Lemma 3.2.

**Lemma 4.1** *For $a \in \mathbb{D}$, $n \in \mathbb{N}^+$ and $\varphi \in \mathcal{U}$, suppose that*

$$\tilde{p}_a(z) = \frac{3(\log\log \frac{4}{1-\overline{\varphi(a)}z})^2}{\log\log \frac{4}{1-|\phi(a)|^2}} - \frac{2(\log\log \frac{4}{1-\overline{\varphi(a)}z})^3}{(\log\log \frac{4}{1-|\varphi(a)|^2})^2}$$

*where $z \in \mathbb{D}$. Then the auxiliary function $\tilde{p}_a$ belongs to the logarithmic Bloch-Orlicz space $\mathcal{B}^{\varphi}_{\log}$ with $\sup_{a\in\mathbb{D}} \|\tilde{p}_a\|_{\mathcal{B}_{\log}} \lesssim 1$.*

**Theorem 4.2** *For $\varphi \in \mathcal{U}$, the weighted composition operator $\psi C_\phi$ is compact on $\mathcal{B}^{\varphi}_{\log}$ if and only if $\psi C_\phi$ is bounded on $\mathcal{B}^{\varphi}_{\log}$,*

$$\lim_{|\phi(z)|\to 1^-} \frac{|\psi'(z)|(2+\log\log \frac{2}{1-|\phi(z)|})}{\varphi^{-1}(\frac{1}{(1-|z|^2)\log \frac{2}{1-|z|}})} = 0 \tag{4.1}$$

*and*

$$\lim_{|\phi(z)|\to 1^-} \frac{|\psi(z)\phi'(z)|\varphi^{-1}(\frac{1}{(1-|\phi(z)|^2\log \frac{2}{1-|\phi(z)|}})}{\varphi^{-1}(\frac{1}{(1-|z|^2)\log \frac{2}{1-|z|}})} = 0. \tag{4.2}$$

*Proof.* Suppose that the weighted composition operator $\psi C_\phi$ is bounded on $\mathcal{B}^{\varphi}_{\log}$ and (4.1) (4.2) hold. Note that $L_1 < \infty$ and $L_2 < \infty$ defined in the proof of Theorem 3.3 (see, (3.2) and (3.3), respectively) by the boundedness of $\psi C_\phi$. For every $\epsilon > 0$, there exists an $0 < r < 1$ such that for $|\phi(z)| > r$,

$$\frac{|\psi'(z)|(2+\log\log \frac{2}{1-|\phi(z)|})}{\varphi^{-1}(\frac{1}{(1-|z|^2)\log \frac{2}{1-|z|}})} < \frac{\epsilon}{2}$$

and

$$\frac{|\psi(z)\phi'(z)|\varphi^{-1}(\frac{1}{(1-|\phi(z)|^2)\log \frac{2}{1-|\phi(z)|^2}})}{(1-|\phi(z)|^2)\varphi^{-1}(\frac{1}{(1-|z|^2)\log \frac{2}{1-|z|}})} < \frac{\epsilon}{2}$$

hold. For a chosen sequence $\{f_n\}_n \subset \mathcal{B}^{\varphi}_{\log}$ that satisfy $\sup_{n\in\mathbb{N}} \|f_n\|_{\mathcal{B}^{\varphi}_{\log}} \leq K$ and $\{f_n\}$ converges to zero uniformly on any compact subsets of the unit disk as $n \to \infty$, where $K$ is a fixed constant. It is sufficient to show that $\lim_{n\to\infty} \|\psi C_\phi f_n\|_{\mathcal{B}^{\varphi}_{\log}} = 0$ by the compactness of $\psi C_\phi$. Note that $\lim_{n\to\infty} f_n(0) = 0$ and $\{f_n'\}$ converges to zero uniformly on any compact subsets of the unit disk. It follows by Proposition 2.3 that

$$\begin{aligned}
\|\psi C_\phi f_n\|_{\mathcal{B}^{\varphi}_{\log}} &= |\psi(0)f_n(\phi(0))| + \sup_{z\in\mathbb{D}} \frac{|(\psi C_\phi f_n)'(z)|}{\varphi^{-1}(\frac{1}{(1-|z|^2)\log \frac{2}{1-|z|}})} \\
&= |\psi(0)f_n(\phi(0))| + \sup_{z\in\mathbb{D}} \frac{|f_n'(\phi(z))\phi'(z)\psi(z) + f_n(\phi(z))\psi'(z)|}{\varphi^{-1}(\frac{1}{(1-|z|^2)\log \frac{2}{1-|z|}})} \\
&\leq |\psi(0)f_n(\phi(0))| + \sup_{\{z\in\mathbb{D}:|\phi(z)|\leq r\}} \frac{|f_n'(\phi(z))\phi'(z)\psi(z) + f_n(\phi(z))\psi'(z)|}{\varphi^{-1}(\frac{1}{(1-|z|^2)\log \frac{2}{1-|z|}})} \\
&\quad + \sup_{\{z\in\mathbb{D}:|\phi(z)|> r\}} \frac{|f_n'(\phi(z))\phi'(z)\psi(z) + f_n(\phi(z))\psi'(z)|}{\varphi^{-1}(\frac{1}{(1-|z|^2)\log \frac{2}{1-|z|}})}
\end{aligned}$$

$$
\begin{aligned}
&\le |\psi(0)f_n(\phi(0))| + L_1 \sup_{\{w\in\mathbb{D}:|w|\le r\}} |f_n(w)| + L_2 \sup_{\{w\in\mathbb{D}:|w|\le r\}} |f_n'(w)| \\
&\quad + \sup_{\{z\in\mathbb{D}:|\phi(z)|>r\}} \frac{\varphi^{-1}(\frac{1}{(1-|\phi(z)|^2)\log\frac{2}{1-|\phi(z)|}})\|f_n\|_{\mathcal{B}^{\varphi}_{\log}}}{\varphi^{-1}(\frac{1}{(1-|z|^2)\log\frac{2}{1-|z|}})} |\phi'(z)\psi(z)| \\
&\quad + \sup_{\{z\in\mathbb{D}:|\phi(z)|>r\}} \frac{(2+\log\log\frac{2}{1-|\phi(z)|})\|f_n\|_{\mathcal{B}^{\varphi}_{\log}}}{\varphi^{-1}(\frac{1}{(1-|z|^2)\log\frac{2}{1-|z|}})} |\psi'(z)| \lesssim \epsilon.
\end{aligned}
$$

Then we conclude that $\psi C_\phi$ is compact on $\mathcal{B}^{\varphi}_{\log}$ by the arbitrariness of $\epsilon > 0$.

Conversely, suppose that $\psi C_\phi$ is compact on $\mathcal{B}^{\varphi}_{\log}$ and hence $\psi C_\phi$ is bounded on $\mathcal{B}^{\varphi}_{\log}$. We prove (4.1) and (4.2) hold as follows. Let $\{z_{n,\log}\}_n$ be a sequence in the unit disk satisfying $\lim_{n\to\infty} |\phi(z_{n,\log})| = 1$. If such sequence does not exist, then the proof is completed.

On the one hand, for each $z \in \mathbb{D}$, we consider the function $\tilde{p}_{\phi(z_{n,\log})}(z)$, where $\tilde{p}_a$ is constructed in Lemma 4.1. Then we have $\tilde{p}'_{\phi(z_{n,\log})}(\phi(z_{n,\log})) = 0$ and

$$
\tilde{p}_{\phi(z_{n,\log})}(\phi(z_{n,\log})) = \log\log\frac{4}{1-|\phi(z_{n,\log})|^2}.
$$

Note that $\{\tilde{p}_{\phi(z_{n,\log})}\}_n$ is bounded uniformly in $\mathcal{B}^{\varphi}_{\log}$ and uniformly converges to zero on any compact subset of the unit disk as $n \to \infty$ by (1.1) . Thus we have $\lim_{n\to\infty} \|\psi C_\phi \tilde{p}_{\phi(z_{n,\log})}\|_{\mathcal{B}^{\varphi}_{\log}} = 0$. It follows that

$$
\lim_{n\to\infty} \frac{|\psi'(z_{n,\log})|}{\varphi^{-1}(\frac{1}{(1-|z_{n,\log}|^2)\log\frac{2}{1-|z_{n,\log}|}})} \le \lim_{n\to\infty} \frac{|\psi'(z_{n,\log})|\log\log\frac{2}{1-|\phi(z_{n,\log})|}}{\varphi^{-1}(\frac{1}{(1-|z_{n,\log}|^2)\log\frac{2}{1-|z_{n,\log}|}})} = 0
$$

and hence

$$
\lim_{|\phi(z)|\to 1^-} \frac{|\psi'(z)|(2+\log\log\frac{2}{1-|\phi(z)|})}{\varphi^{-1}(\frac{1}{(1-|z|^2)\log\frac{2}{1-|z|}})} = 0.
$$

On the other hand, we consider the function $q_{\phi(z_{n,\log})}(z)$, where $q_a$ is constructed in Lemma 3.2. Then we have $q_{\phi(z_{n,\log})}(\phi(z_{n,\log})) = 0$ and

$$
q'_{\phi(z_{n,\log})}(\phi(z_{n,\log})) = \varphi^{-1}(\frac{1}{(1-|\phi(z_{n,\log})|^2)\log\frac{2}{1-|\phi(z_{n,\log})|}}).
$$

Note that $\{q_{\phi(z_{n,\log})}\}_n$ is bounded uniformly in $\mathcal{B}^{\varphi}_{\log}$ and uniformly converges to zero on any compact subset of the unit disk as $n \to \infty$ since

$$
\begin{aligned}
&\varphi^{-1}(\frac{1}{(1-|\phi(z_{n,\log})|^2)\log\frac{2}{1-|\phi(z_{n,\log})|}}) \frac{(1-|\phi(z_{n,\log})|^2)}{|\phi(z_{n,\log})|} \log(1-\overline{\phi(z_{n,\log})}z) \\
&\le \varphi^{-1}(1)\frac{1}{\log\frac{2}{1-|\phi(z_{n,\log})|}} \frac{1}{|\phi(z_{n,\log})|} \log(1-\overline{\phi(z_{n,\log})}z)
\end{aligned}
$$

Thus we have $\lim_{n\to\infty} \|\psi C_\phi q_{\phi(z_{n,\log})}\|_{\mathcal{B}^{\varphi}_{\log}} = 0$. It follows that

$$
\begin{aligned}
&\lim_{n\to\infty} \frac{|\psi(z_{n,\log})\phi'(z_{n,\log})|\varphi^{-1}(\frac{1}{(1-|\phi(z_{n,\log})|^2)\log\frac{2}{1-|\phi(z_{n,\log})|}})}{\varphi^{-1}(\frac{1}{(1-|z_{n,\log}|^2)\log\frac{2}{1-|z_{n,\log}|}})} \\
&\le \lim_{n\to\infty} \|\psi C_\phi q_{\phi(z_{n,\log})}\|_{\mathcal{B}^{\varphi}_{\log}} + \lim_{n\to\infty} \frac{|\psi'(z_{n,\log})|(2+\log\log\frac{2}{1-|\phi(z_{n,\log})|})}{\varphi^{-1}(\frac{1}{(1-|z_{n,\log}|^2)\log\frac{2}{1-|z_{n,\log}|}})} = 0
\end{aligned}
$$

By the same arguments shown in the proof of the boundedness of $\psi C_\phi$ on $\mathcal{B}^{\varphi}_{\log}$, we conclude that

$$\lim_{|\phi(z)|\to 1^-} \frac{|\psi(z)\phi'(z)|\varphi^{-1}(\frac{1}{(1-|\phi(z)|^2 \log\frac{2}{1-|\phi(z)|}})}{\varphi^{-1}(\frac{1}{(1-|z|^2)\log\frac{2}{1-|z|}})} = 0.$$

Combining what we have observed above, we complete the proof. □

# 5 Boundedness and Compactness of Weighted Composition Operators on $\mathcal{B}_{\mu_{\log}}$

Recall that it is proved in Proposition 2.3 that the logarithmic Bloch-Orlicz space is isometrically equal to a $\mu_{\log}-$Bloch space, where

$$\mu_{\log}(z) = \frac{1}{\varphi^{-1}(\frac{1}{(1-|z|^2)\log\frac{2}{1-|z|}})}.$$

Hence, the boundedness and compactness of weighted composition operators on the $\mu_{\log}-$Bloch space $\mathcal{B}_{\mu_{\log}}$ could be naturally given.

**Theorem 5.1** *For $\varphi \in \mathcal{U}$, the weighted composition operator $\psi C_\phi$ is bounded on $\mathcal{B}_{\mu_{\log}}$ if and only if*

$$M_1 := \sup_{z\in\mathbb{D}} \mu_{\log}(z)|\psi'(z)|(2+\log\log\frac{2}{1-|z|}) < \infty$$

*and*

$$M_2 := \sup_{z\in\mathbb{D}} \frac{\mu_{\log}(z)|\psi(z)\phi'(z)|}{\mu_{\log}(\phi(z))} < \infty$$

*hold.*

**Theorem 5.2** *For $\varphi \in \mathcal{U}$, the weighted composition operator $\psi C_\phi$ is compact on $\mathcal{B}_{\mu_{\log}}$ if and only if $\psi C_\phi$ is bounded on $\mathcal{B}_{\mu_{\log}}$,*

$$\lim_{|\phi(z)|\to 1^-} \mu_{\log}(z)|\psi'(z)|(2+\log\log\frac{2}{1-|z|}) = 0$$

*and*

$$\lim_{|\phi(z)|\to 1^-} \frac{\mu_{\log}(z)|\psi(z)\phi'(z)|}{\mu_{\log}(\phi(z))} = 0.$$

# References


1. H. B. Bai, Z. J. Jiang, Generalized weighted composition operators from Zygmund spaces to Bloch-Orlicz type spaces, Appl. Math. Comput. 273 (2016) 89-97.
2. S. Charpentier, Composition operators on Hardy-Orlicz spaces on the ball, Integral Equations Operator Theory 70 (2011) 429-450.
3. C. C. Cowen, B. D. Maccluer, Composition operators on spaces of analytic functions, CRC Press, 1995.

4. J. C. R. Fernández, Composition operators on Bloch-Orlicz type spaces, Appl. Math. Comput. 217 (2010) 3392-3402.

5. J. Giménez, R. Malavé, J. Ramos-Fernández, Composition operators on $\mu-$Bloch type spaces, Rend. Circ. Mat. Palermo 59 (2010) 107-119.

6. Z. Jiang, G. Cao, Composition operator on Bergman-Orlicz space, J. Inequal. Appl. 1 (2009) 1-15.

7. Y. X. Liang, Volterra-type operators from weighted Bergman-Orlicz space to $\beta-$Zygmund-Orlicz and $\gamma-$Bloch-Orlicz spaces, Monatsh. Math. 182 (2017) 877-897.

8. Y. X. Liang, Integral-type operators from $F(p, q, s)$ space to $\alpha$-Bloch-Orlicz and $\beta$-Zygmund-Orlicz spaces, Complex Anal. Oper. Theory 10:8 (2016) 1-26.

9. D. Li, Compact composition operators on Hardy-Orlicz and Bergman-Orlicz spaces, RACSAM 105 (2011) 247-260.

10. P. Lefèvre, D. Li, H. Queffélec, L. Rodríguez-Piazza, Composition operators on Hardy-Orlicz spaces, American Mathematical Society, 207 (2010).

11. P. Lefèvre, D. Li, H. Queffélec, L. Rodríguez-Piazza, Compact composition operators on Bergman-Orlicz spaces, Trans. Amer. Math. Soc. 365 (2013) 3943-3970.

12. P. Lefèvre, D. Li, H. Queffélec, L. Rodríguez-Piazza, Compact composition operators on $H^2$ and Hardy-Orlicz spaces, J. Math. Anal. Appl. 354 (2009) 360-371.

13. K. Madigan, A. Matheson, Compact composition operators on the Bloch space, Trans. Amer. Math. Soc. 347 (1995) 2679-2687.

14. A. K. Sharma, S.D. Sharma, Composition operators on weighted Bergman-Orlicz spaces, Bull. Austral. Math. Soc. 75 (2007) 273-287.

15. J. Xiao, Composition operators associated with Bloch-type spaces, Complex Var. Theor. Appl. 46 (2001) 109-121.

16. R. Yoneda, The composition operators on weighted Bloch space, Arch. Math. (Basel) 78 (2002) 310-317.

17. S. Ye, Multipliers and cyclic vectors on the weighted Bloch space, Math. J. Okayama Univ. 48 (2006), 135-143.

18. K. Zhu, Spaces of holymorphic functions in the unit ball, Graduate Texts in Mathematics 226, Springer, New York, 2005.

19. H. Zhou, Boundedness and compactness of specific weighted composition operators on the Zygmund-Orlicz Space, Journal of Function Spaces, 2022.
