# Supplementary material for: Weighted composition operators on the logarithmic Bloch-Orlicz space
Source: PLoS One. 2024 May 28;19(5):e0303336. doi: 10.1371/journal.pone.0303336 (PMC11132511; doi:10.1371/journal.pone.0303336)
Supplement: S2 File — (DOCX) [file pone.0303336.s002.docx]

This work was supported by the Department of Education of Guangdong Province (Grant Nos. 2022KQNCX121) and there was no additional external funding received for this study.
